# MRPL21-PARP1 axis promotes cisplatin resistance in head and neck squamous cell carcinoma by inhibiting autophagy through the PI3K/AKT/mTOR signaling pathway

**DOI:** 10.1186/s13046-025-03482-9

**Published:** 2025-07-26

**Authors:** Rui Guan, Ce Li, Ruijie Jiao, Jingao Li, Ran Wei, Chen Feng, Shengda Cao, Ye Qian, Jugao Fang, Jun Liu, Wenming Li, Dongmin Wei, Dapeng Lei

**Affiliations:** 1 Department of Otorhinolaryngology, Cheeloo College of Medicine, Qilu Hospital, Shandong University, Jinan, Shandong 250012 China; 2 NHC Key Laboratory of Otorhinolaryngology, Shandong University, 107 West Wenhua Road, Jinan, Shandong 250012 China; 3 Department of Otorhinolaryngology Head and Neck Surgery, Beijing Tongren Hospital, Capital Medical University, Beijing, China; 4 Key Laboratory of Otorhinolaryngology Head and Neck Surgery, Capital Medical University, Ministry of Education, Beijing, China; 5 Department of Otolaryngology-Head & Neck Surgery, West China Hospital, Sichuan University, Chengdu, China

**Keywords:** HNSCC, MRPL21, PARP1, Cisplatin resistance, Autophagy

## Abstract

**Background:**

Head and neck squamous cell carcinoma (HNSCC) constitutes a major clinical challenge that severely affects patient survival. Mitochondrial ribosomal protein (MRP) family plays an important role in energy metabolism by participating in mitochondrial oxidative phosphorylation. However, their roles in HNSCC and the underlying mechanisms are still unclear.

**Methods:**

Single-cell analysis highlighted MRPL21 as a notable biomarker of HNSCC. Human HNSCC tissues, cell lines, and xenograft models in nude mice were used to explore the expression and function of MRPL21. The mass spectrometry was performed to analyze the potential binding targets of MRPL21. In vitro and in vivo experiments were performed to evaluate the effect of MRPL21 on autophagy and cisplatin resistance. The inhibitory actions of siMRPL21 nanodelivery systems on HNSCC progression were also evaluated in vivo.

**Results:**

Clinically, relatively high expression level of MRPL21 was associated with poor prognosis in HNSCC patients, and overexpression of MRPL21 significantly promoted HNSCC tumorigenesis, metastasis, and cisplatin resistance. Mechanistically, MRPL21 upregulated mitochondrial oxidative phosphorylation (OXPHOS) and increased PARylation level, inhibited autophagy through activating the downstream PI3K/AKT/mTOR signaling pathway, and ultimately led to tumor progression and cisplatin resistance in HNSCC.

**Conclusion:**

We conclude that MRPL21 is a novel biomarker and therapeutic target of HNSCC progression and cisplatin resistant, which may provide a new approach for overcoming cisplatin resistance in HNSCC patients.

**Supplementary Information:**

The online version contains supplementary material available at 10.1186/s13046-025-03482-9.

## Background

Head and neck squamous cell carcinoma (HNSCC) represents a significant global health burden, ranking seventh in incidence among all human cancers [1]. Approximately 90% of head and neck cancers are classified as squamous cell carcinomas, among which hypopharyngeal squamous cell carcinoma (HSCC) is associated with patients presenting at an advanced stage and with poor general health [2, 3]. HNSCC treatment is determined by tumor stage, anatomical subsite, and patient-specific factors, with each factor playing a critical role in the decision-making process [4]. With an enhanced comprehension of the molecular mechanisms underlying HNSCC, the use of non-surgical treatments to improve therapeutic outcomes has become a major research focus [5]. Although the combination of targeted therapy and chemotherapy has been shown to improve survival rates in HNSCC patients [6], off-target drug effects and tumor chemoresistance remain significant therapeutic barriers [7]. Thus, identifying novel targets to overcome therapeutic resistance in HNSCC is of importance.

Single-cell RNA (scRNA) sequencing, a technology that enables the amplification and high-throughput sequencing of RNA from individual cells, has been widely applied in multiple studies [8]. Since tumor evolution is driven by the clonal expansion of genetically distinct subpopulations resulting from accumulated mutations, tumor cells exhibit substantial heterogeneity [9], and such heterogeneity is a key driver of tumor invasiveness, metastatic dissemination and therapeutic resistance. However, bulk sequencing methods are unable to effectively resolve cellular heterogeneity, thereby obscuring the clonal deconstruction of cancer evolution and the precise clonal dynamics and evolutionary history of a tumor [10]. scRNA sequencing is therefore a critical player in deciphering tumor heterogeneity and lineage tracing, facilitating the identification of molecular characteristics of tumor cell subpopulations, the elucidation of the tumor microenvironment, and the discovery of potential tumor biomarkers [11].

Nuclear-encoded mitochondrial ribosomal proteins (MRPs) are synthesized in the cytoplasm and assembled with mitochondrial rRNA to form functional ribosomes that are essential for oxidative phosphorylation (OXPHOS) [12]. Metabolic reprogramming represents a hallmark of cancer cells [13], and oncogenic metabolic rewiring often leverages OXPHOS to drive anabolic processes that are essential for tumorigenesis. Accumulating evidence indicates that OXPHOS plays a pivotal role in promoting the progression of HNSCC [14], and that it is correlated with tumor recurrence and cisplatin resistance [15]. MRPs regulate apoptotic processes and maintain cellular energy metabolism [16], both of which are key characteristics of cancer [17]. Additionally, MRPs have also been implicated in tumorigenesis, where they modulate oncogenic signaling networks [18]. For example, MRPL21 promotes the proliferation of hepatocellular carcinoma cells by inducing apoptosis resistance through TP53 mutations [19]. In lung adenocarcinoma (LUAD), MRPL21 has been identified as an independent risk factor and is closely associated with disease progression [20]. However, the functional roles and underlying mechanisms of MRPs in HNSCC remain largely unexplored.

PARP1 is highly expressed and rapidly responsive in cells, exhibiting constitutive expression and rapid activation kinetics. It orchestrates DNA damage response pathways by mediating the post-translational modification of repair proteins [21]. Poly(ADP-ribosyl)ation (PARylation) is a post-translational modification process catalyzed by the PARP (polyADP-ribose polymerase) family of enzymes. Activation of PARylation within cells is involved in numerous processes, including DNA repair, transcriptional regulation, signal transduction, and metabolic modulation [22]. Inhibition of PARP1 has been shown to suppress tumor growth and metastasis [23], enhance the radiosensitivity and chemosensitivity of tumor cells [24], and modulate anti-tumor immune responses [25]. PARP1 further promotes the epithelial-mesenchymal transition (EMT) by coactivating the SMAD4/NF-κB/ZEB1 signaling pathway [26]. Additionally, research has demonstrated that PARP1 is also localized in mitochondria [27], where it plays a crucial role in regulating mitochondrial DNA damage [28] and preserving mitochondrial homeostasis [29].

In this study, scRNA sequencing analysis identified that MRPL21 is an unrecognized driver of HNSCC pathogenesis. MRPL21 affected the mitochondrial OXPHOS process and modulated tumor cell proliferation, metastasis, and autophagy via PARP1 activity and the downstream PI3K/AKT/mTOR signaling pathway, thereby contributing to chemoresistance of HNSCC. MRPL21-targeted nanoparticles also achieved effective inhibition of tumor growth in animal models. Collectively, this study provides new insights into the mechanisms of proliferation and metastasis in head and neck tumors, and highlights MRPL21 as a promising therapeutic target for HNSCC, as well as a potential research direction to enhance chemosensitivity.

## Methods

### Construction of plasmids, siRNA, and stable cell lines

The MRPL21 plasmid, NDUFS1 plasmid, and PARP1 plasmid were obtained from Vigene Biosciences (Shandong, China). MRPL21-targeting siRNA and negative control siRNA, and MRPL21-knockdown and PARP1-knockdown lentiviruses were from GenePharma (Shandong, China). Plasmids and siRNAs were transiently transfected into HNSCC cells using the jetPRIME reagent (101000046, Polyplus). All transfections were carried out strictly following the manufacturer’s protocols. Target sequences for shRNA and siRNA are listed in Supplementary Table 1.

### RNA-sequencing (RNA-seq) of stably transfected cells and analysis

Recombinant MRPL21 lentiviruses were transfected into FaDu cells according to the manufacturer’s instructions for 72 h. Stably transfected cells were selected using puromycin (2 µg/mL; ST551, Beyotime, China). Western blot and quantitative reverse-transcription PCR (qRT-PCR) analyses were performed to validate the transfection efficiency. The stably transfected cells with the highest efficiency were subsequently selected for RNA sequencing and further experiments. Three biological replicates of each cell line were prepared and sent to Genechem (Shanghai, China) for analysis.

### Clinical specimens

Hypopharyngeal samples, including normal and tumor tissues, were collected from patients undergoing surgery under sterile conditions at the Department of Otolaryngology, Qilu Hospital, Shandong University. This study was conducted in accordance with the protocol approved by the Ethics Committee of Qilu Hospital, Shandong University, and signed informed consent was obtained from all patients. Following tissue dissection, the samples were confirmed by diagnostic pathology and stored in liquid nitrogen for RNA extraction.

### Bioinformatics analysis

The analysis and visualization of different genes and the correlation between gene expression and patient overall survival in The Cancer Genome Atlas (TCGA) database were conducted using GEPIA (http://gepia.cancer-pku.cn/) [30] and the bioinformatics platform at https://www.bioinformatics.com.cn [31].

### Cell culture

Detroit562, Cal27, TU686, and FaDu cell lines were obtained from the American Type Culture Collection (ATCC, Manassas, USA); and FaDu/DDP cell lines were purchased from Hefei Wuhan Biotechnology Co., Ltd. All cell lines used in this study were authenticated by short tandem repeat (STR) profiling and tested negative for *Mycoplasma* contamination. TU686 cells were cultured in RPMI 1640 medium (22400089, Gibco), while all other cells were maintained in DMEM (10569044, Gibco) supplemented with 10% fetal bovine serum (FBS, HyClone) and 1% penicillin-streptomycin solution (C0222, Beyotime) at 37 °C in a humidified atmosphere containing 5% CO_2_.

### Western blot analysis and nuclear and cytoplasmic protein extraction

Cells were lysed using RIPA lysis buffer (Solarbio Biotechnology, Beijing, China) supplemented with PMSF (ST505, Beyotime) and complete protease inhibitor cocktail (EASYpacks, 04693132001, Roche). Total protein extracts were quantified and separated in 4–12% or 4–20% precast gels (Yeasen Biotechnology, Shanghai, China). Proteins were transferred onto NC membranes (Millipore, Billerica, MA, USA). The membranes were blocked in TBST containing 5% non-fat milk for 1 h and incubated with primary antibodies at 4 °C overnight, followed by horseradish peroxidase (HRP)-conjugated goat anti-rabbit or anti-mouse IgG secondary antibody (Immunoway, Plano, TX, USA) for 1 h at room temperature. Protein bands were revealed with an ECL Advance Western Blotting Detection Kit (P0018AS, Beyotime) and visualized with a Tanon 4600 chemiluminescence image system (Tanon, Shanghai, China). Band intensities were analyzed using ImageJ software (version 1.52, NIH, USA). The primary antibodies used are listed in Supplementary Table 2.

Nuclear and cytoplasmic proteins were extracted using a Nuclear and Cytoplasmic Protein Extraction Kit (P0027, Beyotime) according to the manufacturer’s instructions, and samples were analyzed by western blotting.

### RNA isolation and real-time quantitative reverse-transcription PCR analysis

Total RNA was extracted from samples using the RNAsimple Total RNA Kit (DP419, TIANGEN) according to the manufacturer’s instructions. Equal amounts of RNA were reverse-transcribed into cDNA using the SPARKscript II All-in-one RT SuperMix for qPCR (AG0305, SparkJade). RT-qPCR was performed using 2×SYBR Green qPCR Mix (AH0104, SparkJade) on a LightCycler^®^ 480 Instrument II (Roche). Relative gene expression was calculated with the 2^−∆∆Ct^ method and β-actin was used as the reference gene for data normalization. The primer sequences employed in this study are listed in Supplementary Table 3.

### Cell viability assays

Cells (3 × 10^3^ cells/well) were seeded in 96-well plates and cultured for 24 h. Then a 10-µL solution from a Cell Counting Kit-8 (CCK8) (CK04, Dojindo) was added to each well according to the protocol of the manufacturer, followed by incubation at 37 °C for 1 h. The optical density (OD) values were measured using a spectrometer at 450 nm and 650 nm, with each well being independently read three times. Cell proliferation was evaluated using a BeyoClick™ EdU Cell Proliferation Kit with Alexa Fluor 594 (C0078S, Beyotime, Shanghai, China) following the manufacturer’s instructions. After washing with PBS, the cells were incubated with EdU solution for 2 h, and the cell nuclei were then stained with Hoechst 33342 for observation.

### Colony formation assay

For the colony formation assay, 1000 cells per well were seeded in 6-well plates and cultured at 37 °C under 5% CO_2_ for 14 days. The cell colonies were then fixed with 4% paraformaldehyde and stained with crystal violet (0.5% solution, Beyotime) and counted.

### Cell cycle assay

Cells (5 × 10^6^ cells/well) were seeded in a 6-well plate and collected by trypsinization and washed twice in cold PBS. Cells were then fixed with ice-cold 75% ethanol overnight at 4 °C. Following fixation, the cell suspension was incubated with 20 µL of RNase A (BestBio, Shanghai, China) for 30 min at 37 °C and then stained with 400 µL of propidium iodide (BestBio) for 30 min at 4 °C. Flow cytometric analyses and measurements were performed using a BD Accuri™ C6 Plus flow cytometer (BD Bioscience, Mountain View, USA).

### Wound healing and transwell assays

For the wound healing assay, cells were plated in 12-well plates to form a full confluent monolayer. A linear wound was created by scratching with a sterile 200 µL pipette tip, and the intercellular distance at each timepoint was measured using a Lionheart automated microscope (BioTek, USA).

For the Transwell migration assay, 3 × 10^4^ cells suspended in 200 µL of serum-free medium were plated onto the upper Transwell chamber containing an 8.0 μm pore-sized polycarbonate membrane (353097, BD Falcon). The chamber was then placed in a 24-well plate containing 500 µL of medium supplemented with 10% fetal bovine serum. After the incubation period, non-migrated cells were removed using a cotton swab, and the migrated cells adhering to the bottom surface of the membrane were fixed with 4% paraformaldehyde, stained with crystal violet, and counted under a light microscope.

### Protein mass spectrometry and co-immunoprecipitation assays

Mass spectrometry‑based proteomics and co-immunoprecipitation (Co-IP) was performed with the Immunoprecipitation Kit-Immunomagnetic Beads Protein A/G (C0951, Bioss Biotechnology, China) according to the manufacturer’s instructions. Cells were lysed in NP40 cell lysis buffer containing PMSF (1 mM). For protein mass spectrometry, the lysate was incubated overnight at 4 °C with anti-Flag antibody, and then incubated with the immunomagnetic beads for 2 h at 4 °C. The complex was sent for protein identification by liquid chromatography with tandem mass spectrometry (LC-MS/MS). For co-immunoprecipitation assays, the lysate was centrifuged to obtain 500 µg of total protein. Subsequently, 5 µg of specific antibodies was mixed with the protein sample and incubated overnight at 4 °C. The pre-bound complex was incubated with the immunomagnetic beads for 2 h at 4 °C. After washing, the immunomagnetic bead antibody-antigen complex was eluted by adding 1× SDS-PAGE loading buffer, followed by heating at 100 °C for 10 min. The samples were cooled and subjected to western-blot analysis.

### Apoptosis assay

After 24 h of cisplatin treatment, cells were harvested and resuspended in Binding Buffer at a concentration of 1 × 10^6^ cells/mL. The cells were incubated with annexin V-APC and 7-AAD (Annexin V-APC/7-AAD apoptosis kit, AP105, MULTI SCIENCES) for 10 min at room temperature in the dark following the manufacturer’s instructions. Afterward, the stained cells were subjected to flow cytometric analysis. For the TUNEL assay, cells were fixed with 4% paraformaldehyde for 30 min and incubated with detection buffer for 1 h, then washed with PBS and prepared for observation under a fluorescence microscope.

### Immunofluorescence staining

Cells were washed with PBS three times and fixed with 4% paraformaldehyde and then permeabilized with 0.1% Triton X-100. After blocking with goat serum for 1 h at room temperature, the cells were incubated with two different primary antibodies at 4 ℃ overnight. The cells were then incubated for 1 h with Alexa Fluor^®^ 488/594 anti-rabbit IgG secondary antibodies and Alexa Fluor^®^ 488/594 anti-mouse IgG secondary antibodies. Finally, nuclei were stained with DAPI (Beyotime Biotechnology, Shanghai, China).

### Animal models

Four-week-old male BALB/c nude mice were purchased from GemPharmatech (Nanjing, China) and housed under standard specific-pathogen-free (SPF) conditions. Mice were randomly divided into different groups (*n* = 6 mice/group). Approximately 1 × 10^7^ cells transfected with GFP-luc lentivirus (GenePharma, Shandong, China) were subcutaneously inoculated into the mice to establish the xenograft model. Tumor volume was measured every three days using a caliper and calculated according to the standard formula 0.5 × length × width^2^. For bioluminescence imaging, the mice were intraperitoneally injected with d-luciferin sodium salt (40901ES01, Yeasen) (150 mg/kg), and the bioluminescence of tumors was detected using an IVIS Spectrum instrument (Perkin Elmer, USA). The mice were euthanized and tumor tissues were weighed and collected for immunohistochemical (IHC) staining.

For the metastasis model, approximately 3 × 10^6^ cells suspended in 150 µl of PBS were injected into the tail veins of mice. After six weeks, lung bioluminescence was evaluated using the IVIS Spectrum instrument. The mice were then euthanized and lung tissues were collected for hematoxylin and eosin (H&E) staining.

### Immunohistochemistry and IHC scoring

Tumor samples were fixed, paraffin-embedded, and sectioned into 4 μm thick slices. Tissue slides were prepared following standard procedures, including deparaffinization, dehydration and antigen retrieval. Non-specific binding was blocked using goat serum. Slides were then incubated with primary antibody overnight at 4 °C, followed by incubation with secondary antibodies for 1 h at room temperature. Finally, DAB staining, hematoxylin redyeing and hydrochloric acid alcohol differentiation were performed to enhance nuclear contrast. The stained slides were scanned by whole slide imaging (WSI) (Servicebio, Wuhan, China), and the images were analyzed using CaseViewer software. For scoring, we categorized the proportion of stained-positive cells into four groups: 1 (0–25%), 2 (26–50%), 3 (51–75%), and 4 (76–100%). Staining intensity was scored as follows: 0 (no appreciable staining); 1 (weak intensity, light yellow); 2 (moderate intensity, yellow‒brown); and 3 (strong intensity, brown). The final staining index was calculated as the scope of stained-positive cells score × the staining intensity score.

### Intracellular ATP levels

Intracellular ATP levels were measured using ATP Assay Kits (Beyotime) according to the manufacturer’s instructions. Relative luminescence units (RLUs) were measured with a single-tube luminometer (Promega, Madison, USA).

### Assay of mitochondrial complex I activity

Mitochondrial complex I activity was measured using a CheKine™ Micro Mitochondrial complex I Activity Assay Kit (Abbkine, Wuhan, China) following the manufacturer’s instructions. Complex I in the mitochondria was extracted from 5 × 10^6^ cells, and the enzyme activity of complex I was assessed by measuring nicotinamide adenine dinucleotide (NADH) oxidation over 2 min at 340 nm.

### Determination of mitochondrial membrane potential

For the mitochondrial membrane potential (Δψm) assay, treated cells were stained with the potentiometric dye tetramethylrhodamine ethyl ester (500 nM TMRE; C2001S, Beyotime) at 37 °C for 20 min. After washing, staining was visualized using a confocal scanning microscope.

### Determination of mitochondrial reactive oxygen species (ROS)

Mitochondrial superoxide levels were quantified using MitoSOX™ Red (M36008, Invitrogen). Briefly, 1 mL of MitoSOX™ (5 µM) was added to the cells and the solution was incubated for 10 min at 37 °C in the dark.

### Determining the NAD+/NADH ratio

The NAD+/NADH ratio was measured using an NAD+/NADH assay WST-8 Kit (S0175, Beyotime) according to the manufacturer’s instructions.

### nHAp-PLL-siMRPL21 complex synthesis and characterization

Poly-L-lysine (PLL, 0.1%) (P303210, Aladdin) and nano-hydroxyapatite (nHAp, 1 mg/mL, diameter < 100 nm) (H106378, Aladdin) were mixed at various ratios (ranging from 1:16 to 8:1) and reacted through continuous shaking at 4 °C for 24 h. The resultant complex was centrifuged at 10,000 × *g* for 10 min to isolate the nHAp-PLL compound. The compound was then resuspended in HBS buffer, ultrasonicated for 15 min to ensure particle dispersion, and sterilized by filtration through a 0.22-µm filter. The sterilized nHAp-PLL was stored at 4 °C for subsequent applications. The nHAp-PLL compound was gently mixed with GP-ESC siMRPL21 from GenePharma (Shandong, China) at a mass ratio of 30:1 at room temperature for 1 h to form the nHAp-PLL-siMRPL21 nanocarrier system. The size and surface charge of the particles were characterized by dynamic light scattering (DLS). The morphology, structure, and chemical composition of the nanoparticles were further examined by transmission electron microscopy (TEM), field-emission scanning electron microscopy (FE-SEM), and energy-dispersive spectroscopy (EDS).

### Statistical analysis

All experiments were independently repeated at least three times unless otherwise specified. Quantification of the results was performed by blinded observers and is presented as the mean ± SEM. Statistical analysis was conducted using GraphPad Prism 10.1.2 (GraphPad Software, San Diego, CA, USA). Intergroup differences were assessed with Student’s *t* test. Data met a normal distribution and *P* < 0.05 was considered statistically significant. Significance levels were denoted as follows: * *P* < 0.05, ** *P* < 0.01, *** *P* < 0.001, **** *P* < 0.0001.

## Results

### Single-cell RNA sequencing reveals MRPL21 as a prognostic biomarker in HNSCC

**Fig. 1:**
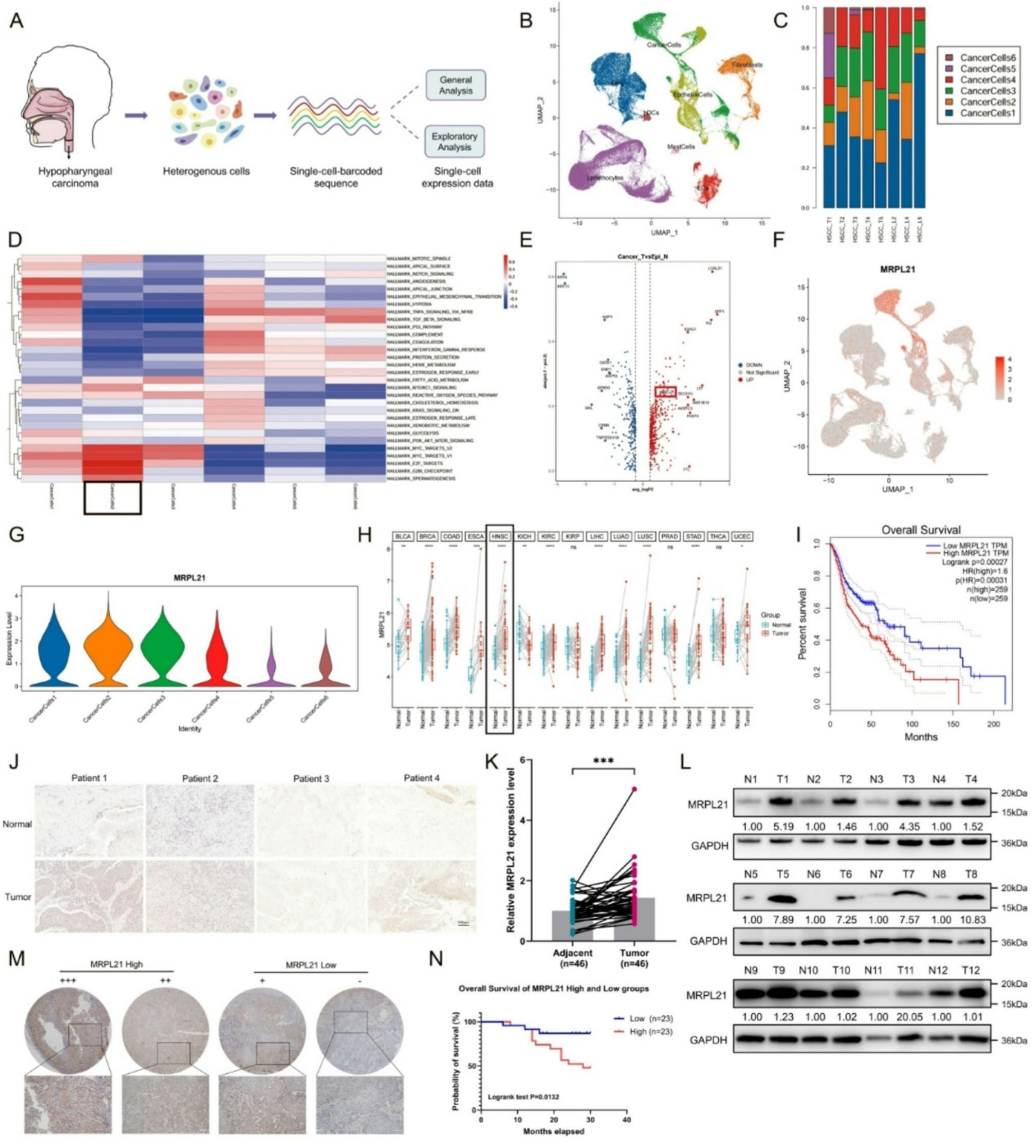
Comprehensive analysis of MRPL21 in cancer cells and tissues. (**A**) Overview of the scRNA-seq process for human hypopharynx tumor tissues. (**B**) UMAP plots illustrating the distribution of different cell types in the tumor microenvironment. (**C**) Bar charts presenting the proportions of each cancer cell subtype across samples. (**D**) Heatmap highlighting the top hallmarks associated with six subtypes. (**E**) Volcano plot illustrating differential gene expression between cancer cells and epithelial cells. (**F**) UMAP plot showing the distribution of MRPL21 expression in different cell types. (**G**) Violin plots quantifying MRPL21 expression levels in different cancer cell subtypes. (**H**) MRPL21 expression detection in multiple tumor types. (**I**) Survival analysis assessing the prognostic significance of MRPL21 in HNSCC patients. (**J**) Immunohistochemical staining depicting MRPL21 expression levels in adjacent normal and tumor tissues. Scale bars, 100 μm. (**K**) Quantification of MRPL21 mRNA expression levels in 46 pairs of HNSCC samples. (**L**) Western blot analysis evaluating the protein expression levels of MRPL21 in HNSCC tissues. (**M**) Representative immunohistochemical staining images showcasing MRPL21 expression in tumor tissues with relative high and low expression intensities. Scale bars, 100 μm. (**N**) Survival analysis correlating MRPL21 expression with clinical outcomes in HNSCC patients

In our preliminary study, we collected paired normal adjacent tissues, primary tumors, and metastatic lymph nodes from five HSCC patients for scRNA-seq analysis (Fig. 1A). Cell clusters were subsequently annotated using lineage-specific gene markers and epithelial cell populations that were specifically present in tumors and metastatic lymph node tissues were designated as “cancer cells” (Fig. 1B). We further subdivided the cancer cell population into six subclusters that exhibited distinct distribution patterns and proportions within the primary tumors and metastatic lymph node tissues (Fig. 1C; Supplementary Fig. S1B). To further elucidate the status and characteristics of each subcluster, we conducted pseudo-time trajectory analysis and assessed the dynamic gene-expression patterns and potential transcriptional regulators across each subgroup (Supplementary Fig. S1C–E). Based on the subcluster-specific top 10 differentially expressed genes (Supplementary Fig. S1F), we performed functional enrichment analysis that included KEGG pathways and hallmark gene sets, revealing subcluster-specific functional characteristics and biological processes (Fig. 1D; Supplementary Fig. S1G). Subpopulation 2 showed marked enrichment of cell cycle, E2F targets, and G2M checkpoints, consistent with a pro-proliferative phenotype. Intersection analysis of highly expressed genes in three different tissues and subpopulations of cancer cells in subpopulation 2 yielded nine candidate genes (Supplementary Fig. S1H). Given the critical role of mitochondrial metabolism in cancer, we explored the expression of the MRPL21 gene in different tissue types and various cell subpopulations (Fig. 1E; Supplementary Fig. S1I). Our results demonstrated that MRPL21 was predominantly expressed in cancer cells, with the highest proportion in cancer cell subpopulation 2 (Fig. 1F–G; Supplementary Fig. S1J–K). MRPL21 exhibited high expression levels across multiple cancer types, including HNSCC (Fig. 1H). Survival analysis further revealed that elevated MRPL21 expression was significantly associated with reduced overall survival and 5-year survival in HNSCC patients (Fig. 1I; Supplementary Fig. S1L). Subsequently, we validated the expression of MRPL21 in clinical samples, and detected increased transcript and protein levels of MRPL21 in HNSCC tumor tissues (Fig. 1J–L). Patients were stratified into high and low MRPL21-expression groups based on RNA and protein levels (Fig. 1M), and our analysis confirmed a markedly worse prognosis and shorter survival duration in the high-expression group (Fig. 1N).

### MRPL21 promotes HNSCC progression by enhancing cell proliferation and migration and facilitating the EMT process

**Fig. 2:**
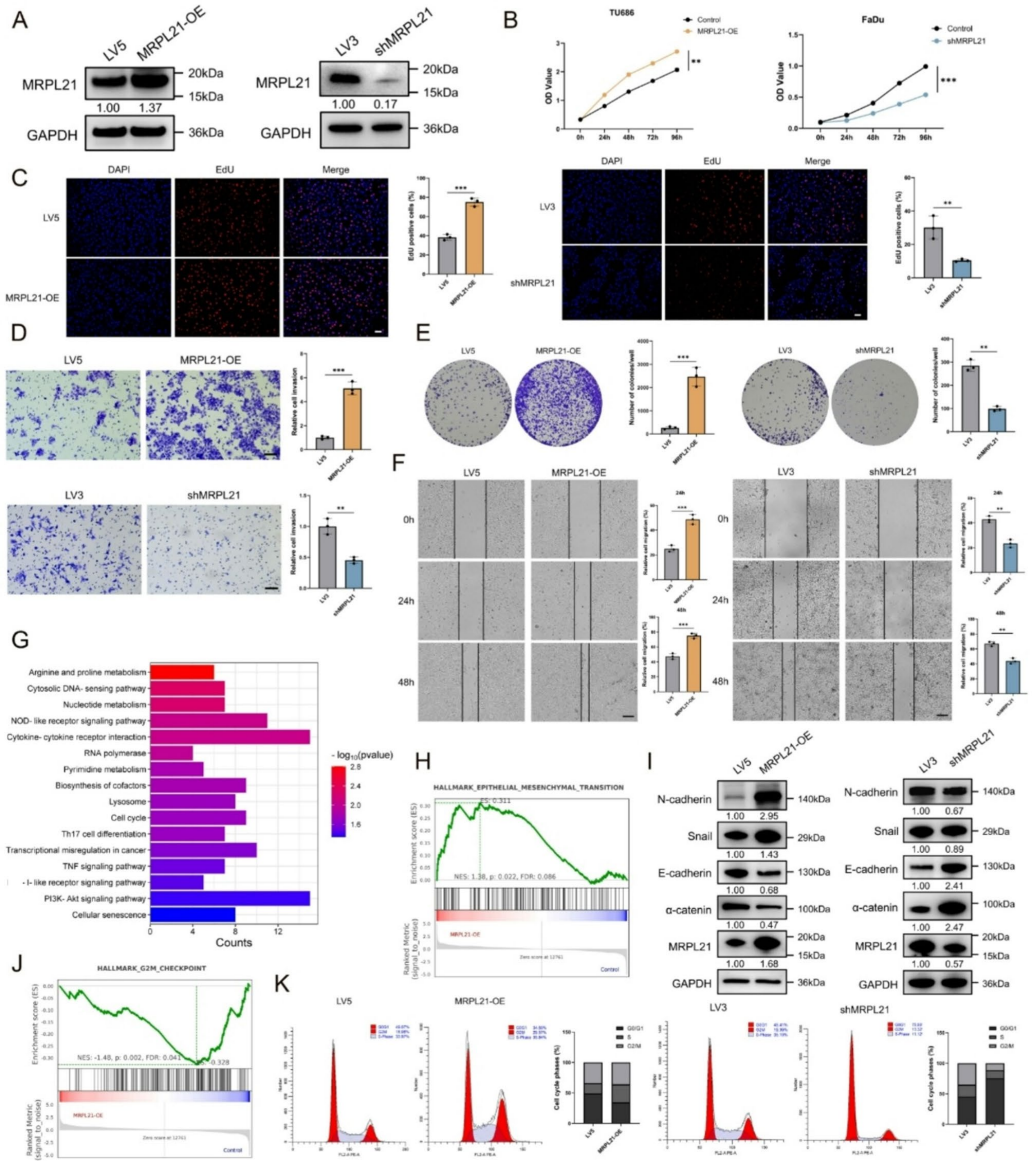
MRPL21 promotes tumor progression and the EMT in HNSCC. (**A**) Western blot analysis confirming the efficiency of MRPL21 lentivirus. (**B**) CCK8 assay evaluating cell viability after MRPL21 overexpression and knockdown. (**C**) EdU incorporation assays and statistical analysis demonstrating the proliferative capacity of FaDu and Tu686 cells. Scale bars, 50 μm. (**D**) Transwell assays demonstrating the impact of MRPL21 on cell invasive ability. Scale bars, 100 μm. (**E**) Colony formation assays assessing the effect of MRPL21 on cell proliferation. (**F**) Wound healing assays showing cell migratory capability. Scale bars, 200 μm. (**G**) KEGG analysis of DEGs. (**H**) GSEA plots showing the enrichment scores for HALLMARK_EMT pathway. (**I**) Western blot analysis demonstrating the relationship between MRPL21 and EMT markers. (**J**) GSEA plots showing the enrichment scores for HALLMARK_G2M checkpoint pathway. (**K**) Flow-cytometric analysis illustrating the distributional percentage of cells in different cell cycle phases

To further elucidate the role of MRPL21 in the development of HNSCC, we initially evaluated the basal expression levels of MRPL21 in various HNSCC cell lines, revealing high expression in the hypopharyngeal carcinoma cell line FaDu and relatively low expression in the laryngeal carcinoma cell line TU686 (Supplementary Fig. S2A). Based on this finding, we performed MRPL21 knockdown in FaDu cells and MRPL21 overexpression in TU686 cells using a GFP-tagged lentiviral system. The efficiencies of both knockdown and overexpression were confirmed by GFP fluorescence detection, qRT-PCR for mRNA levels (Supplementary Fig. S2B–C), and western blot analysis for protein expression (Fig. 2A). We subsequently examined cell proliferation using CCK8 (Fig. 2B) and EdU assays (Fig. 2C), evaluated the impact on tumor cell invasion and migration using Transwell (Fig. 2D) and wound healing assays (Fig. 2F), and investigated changes in clonogenic proliferation using colony formation assays (Fig. 2E). The results showed that overexpression of MRPL21 significantly promoted proliferation, migration, and clonogenic ability in FaDu cells, while MRPL21 knockdown suppressed these processes in TU686 cells.

To further elucidate the specific mechanisms by which MRPL21 exerts its oncogenic effects and the potential pathways involved, transcriptomic profiling of MRPL21-overexpressing cells identified 250 upregulated and 336 downregulated genes (Supplementary Fig. S2D). Pathway enrichment analysis of differentially expressed genes (DEGs) demonstrated that key pathways such as cell cycle, TNF signaling pathway, PI3K-AKT signaling pathway, and cellular senescence were significantly enriched (Fig. 2G). Gene ontology (GO) analysis highlighted that MRPL21 was involved in the regulation of various enzyme and receptor ligand activities within the cell (Supplementary Fig. S2E). Furthermore, gene set enrichment analysis (GSEA) of hallmark pathways indicated that MRPL21 overexpression was associated with angiogenesis, P53-related pathway, EMT process, and other tumor malignancy-related pathways (Fig. 2H; Supplementary Fig. S2F). Assessment of EMT-related markers confirmed that MRPL21 overexpression downregulated epithelial markers and upregulated mesenchymal proteins, while its knockdown had the opposite effect (Fig. 2I). Additionally, MRPL21 influenced DNA repair and cell division through the G2M checkpoint, implicating its dual roles in tumor initiation and progression (Fig. 2J). Flow-cytometric analysis corroborated these findings, showing that MRPL21 overexpression increased the proportions of cells in the S and G2-M phases, whereas its knockdown exhibited the opposite effect, consistent with its pro-proliferative role (Fig. 2K).

### MRPL21 interacts with PARP1 to regulate HNSCC progression and the EMT

**Fig. 3:**
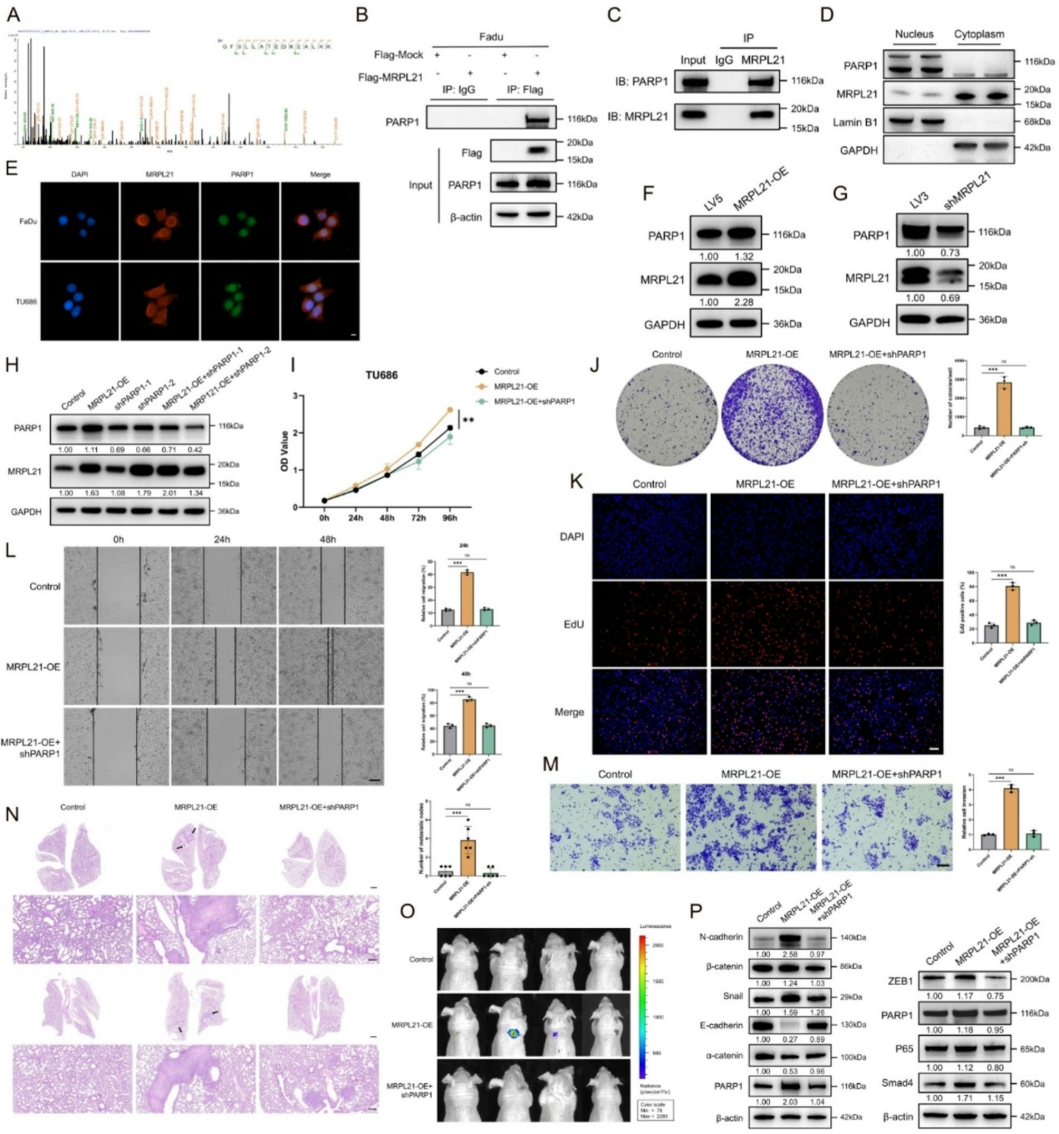
Analysis of MRPL21 interactions with PARP1 and effects on HNSCC progression and the EMT. (**A**) Protein alignment and sequence logo of PARP1 in MRPL21 protein mass spectrometric experiment. (**B**) IP assay showing the interaction between PARP1 and Flag-tagged MRPL21. (**C**) IP assay showing the interaction between MRPL21 and PARP1. (**D**) Subcellular fractionation of the distribution of PARP1 and MRPL21 in nuclear and cytoplasmic fractions. (**E**) Fluorescence microscopic images of the co-localization of MRPL21 and PARP1 in cells. Scale bars, 2 μm. (**F**) Western-blot analysis depicting the expression levels of PARP1 after MRPL21 overexpression. (**G**) Western blot analysis showing the expression levels of PARP1 after MRPL21 knockdown. (**H**) Western-blot analysis illustrating the expression levels of PARP1 overexpression. (**I**) CCK8 assay of cells. (**J**) Colony formation assays demonstrating cell proliferation. (**K**) EdU incorporation assay of cells. Scale bars, 50 μm. (**L**) Wound healing assays of cell migratory capability. Scale bars, 200 μm. (**M**) Transwell assays demonstrating cell invasive ability. Scale bars, 100 μm. (**N**) Hematoxylin and eosin (**H**&**E**) staining of mouse lungs. Scale bars, 1000 μm (upper), 100 μm (below). (**O**) Bioluminescence imaging of lung metastatic tumors. (**P**) Western blot analysis showing the expression levels of EMT markers and downstream proteins

To further investigate the underlying mechanisms of MRPL21’s tumor-promoting function, we transfected FaDu cells with a Flag-tagged MRPL21 overexpression plasmid or its corresponding control plasmid, performed an immunoprecipitation (IP) experiment using Flag IP antibody to identify potential interacting proteins (Supplementary Fig. S3A), and demonstrated that PARP1 protein was significantly enriched in the overexpression group compared to the control group (Fig. 3A; Supplementary Fig. S3B). Given its critical role in DNA damage repair and tumorigenesis, we examined the interaction between the two proteins via exogenous co-IP in MRPL21-overexpressing cells (Fig. 3B) and an endogenous Co-IP experiment using MRPL21 IP antibody in native HNSCC cells, and this analysis confirmed specific binding between MRPL21 and PARP1 (Fig. 3C). Subcellular fractionation further revealed nuclear co-localization of MRPL21 and PARP1 (Fig. 3D), which was corroborated by immunofluorescence showing their nuclear overlap across HNSCC cell types (Fig. 3E).

To investigate whether MRPL21 affected the basal expression of PARP1, we examined PARP1 expression levels in cell lines with MRPL21 knockdown and overexpression. The results demonstrated that PARP1 expression increased upon MRPL21 overexpression and decreased upon MRPL21 knockdown (Fig. 3F–G), suggesting that MRPL21 regulates tumor progression by binding to PARP1 and modulating its expression. To further explore the oncogenic effects of MRPL21, we stably knocked down PARP1 in MRPL21-overexpressing cells and assessed its impact on MRPL21-driven phenotypes (Fig. 3H; Supplementary Fig. S3C). CCK8, EdU, and colony formation assays showed that PARP1 depletion attenuated MRPL21-driven proliferation (Fig. 3I–K). Additionally, wound healing and Transwell assays indicated that PARP1 knockdown reversed the MRPL21-mediated enhancement of tumor cell migration and invasion (Fig. 3L and M). Given that previous studies have shown PARP1 plays a regulatory role in the EMT process in tumors, we sought to determine whether PARP1 is involved in MRPL21-mediated regulation of the EMT in HNSCC. We established a lung metastasis model in nude mice via intravenous injection of three groups of cells. We observed that MRPL21 overexpression significantly increased metastatic lung nodules compared to controls, and the effect was rescued by PARP1 knockdown (Fig. 3N–O). Furthermore, metastases from MRPL21-overexpressing tumors exhibited elevated levels of EMT markers and PARP1 downstream effectors, which were normalized upon PARP1 silencing (Fig. 3P–Q).

### MRPL21 regulates cell proliferation, migration, and tumor growth in vitro and in vivo through PARP1

**Fig. 4:**
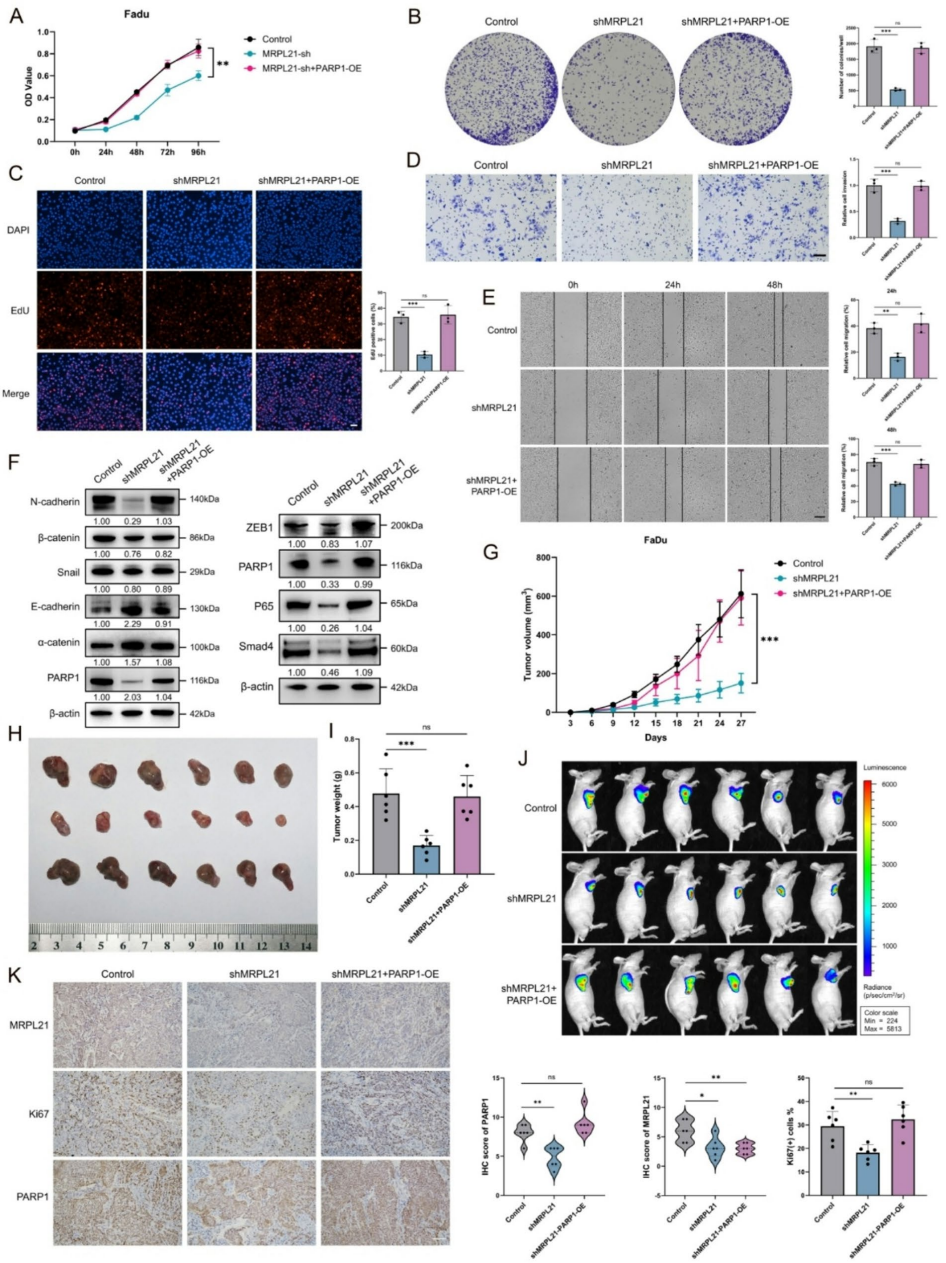
Effects of MRPL21 and PARP1 on cell proliferation, migration, and tumor growth in vitro and in vivo. (**A**) CCK8 assay of cells after MRPL21 knockdown and PARP1 overexpression. (**B**) Colony formation assays of cellular proliferation. (**C**) EdU incorporation assay and statistical analysis. Scale bars, 50 μm. (**D**) Transwell assays demonstrating the effects of MRPL21 and PARP1 on cell invasive ability. Scale bars, 100 μm. (**E**) Wound healing assays depicting cell migratory ability. Scale bars, 200 μm. (**F**) Western blot analysis showing the expression levels of EMT markers and downstream proteins. (**G**) Tumor volume measurements in xenograft mouse model treated with control, shMRPL21, or shMRPL21 + PARP1-OE cells. (**H**) Photographs of tumor tissues harvested from the xenograft mouse models. (**I**) Tumor weight measurements in xenograft mouse models. (**J**) Bioluminescence imaging of lung metastatic tumors. (**K**) Immunohistochemical staining of xenograft tumors and positive correlation statistics. Scale bars, 100 μm

We performed cellular function validation experiments in MRPL21 knockdown and PARP1-overexpressing cells (Supplementary Fig. S3D–E). CCK8, EdU, and colony formation assays demonstrated that PARP1 overexpression significantly reversed the inhibitory effects of MRPL21 knockdown on tumor cell proliferation (Fig. 4A–C). Transwell and wound healing assays showed that compared to the MRPL21 knockdown group, cell migration and invasion were significantly enhanced in the PARP1-overexpressing group (Fig. 4D and E). We also investigated the expression of EMT markers and downstream proteins related to PARP1 in MRPL21 knockdown and PARP1-overexpressing groups (Fig. 4F). To investigate the in vivo regulatory role of MRPL21 in tumor progression, we established an HNSCC xenograft model by subcutaneously injecting cells harboring knockdown of MRPL21 and PARP1-overexpressing cells, as well as control cells, into nude mice. In vivo imaging and tumor measurements indicated that compared to the control group, tumor growth rate, volume, and weight were significantly reduced in the MRPL21-knockdown group, while no significant differences were observed in the PARP1-overexpressing rescue group (Figs. 4G–J). Immunohistochemical (IHC) analysis of the xenograft tumors revealed that the expression levels of the proliferation marker Ki67, as well as of MRPL21 and PARP1, were significantly downregulated in the knockdown group, with PARP1-overexpressing rescue tumors exhibiting expression patterns similar to those of the controls (Fig. 4K).

### PARP1 is involved in the regulation of cisplatin resistance in HNSCC by MRPL21 in vitro and in vivo

**Fig. 5:**
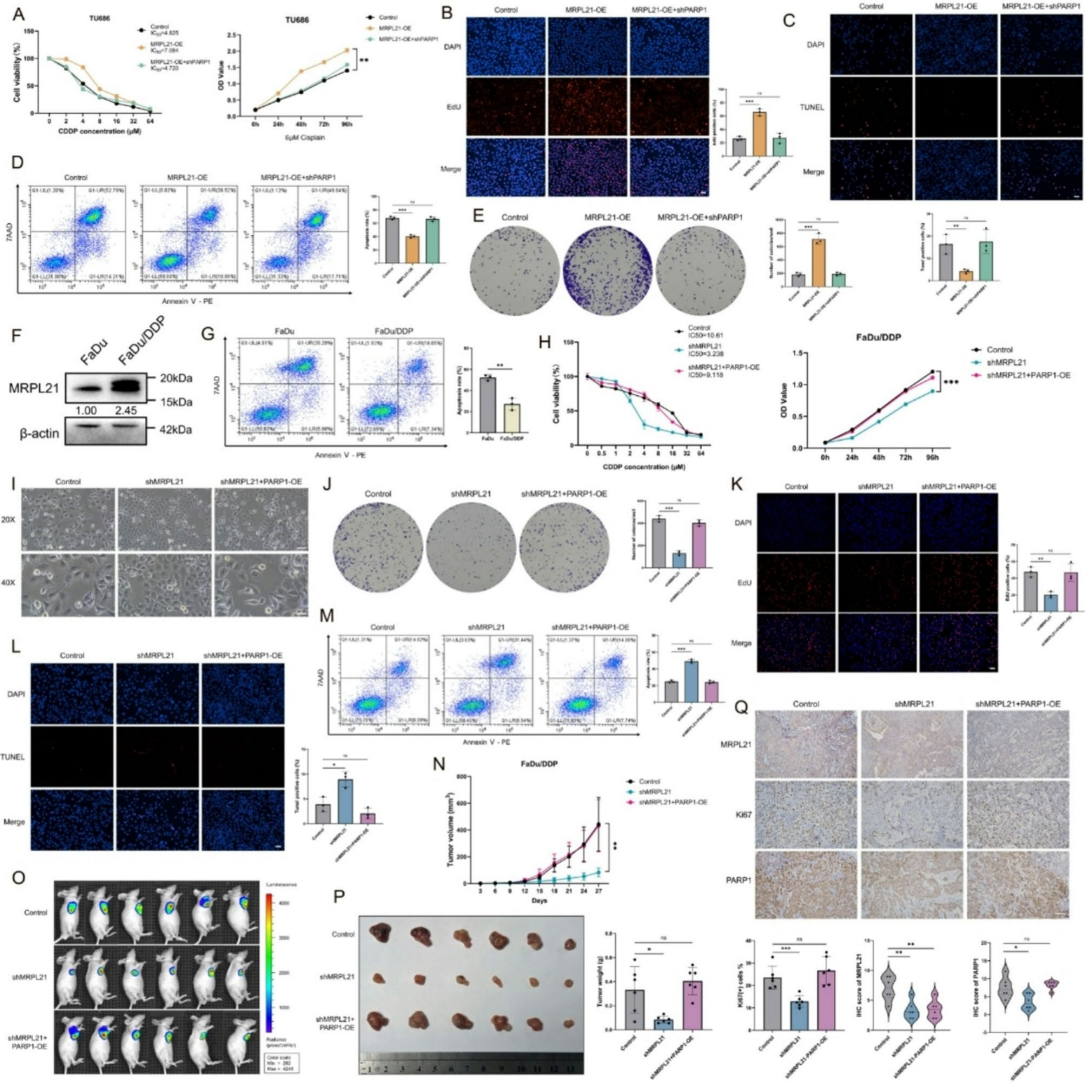
Effect of MRPL21 on cisplatin resistance in HNSCC. (**A**) Cell viability assay and IC50 assay of cisplatin-treated TU686 cells. (**B**) EdU incorporation assays showing DNA replication in treated cells. Scale bars, 50 μm. (**C**) TUNEL assays demonstrating apoptosis in treated cells; TUNEL-positive cells are shown in red. Scale bars, 50 μm. (**D**) Flow- cytometric analysis of DNA damage using 7-AAD staining. (**E**) Colony formation assays of cisplatin-treated TU686 cells. (**F**) Western-blot analysis of the expression levels of MRPL21 in FaDu/DDP cells. (**G**) Flow-cytometric analysis of apoptosis in FaDu/DDP cells and controls. (**H**) Cell viability assay and IC50 assay of cisplatin-treated FaDu/DDP cells. (**I**) Morphologic changes in FaDu/DDP cells. Scale bars, 50 μm (upper), 25 μm (below). (**J**) Colony formation assays demonstrating cellular proliferation. (**K**) EdU incorporation assays showing DNA replication. Scale bars, 50 μm. (**L**) TUNEL assays demonstrating apoptosis. Scale bars, 50 μm. (**M**) Flow-cytometric analysis of apoptosis in shMRPL21 and shMRPL21 + PARP1-OE FaDu/DDP cells. (**N**) Tumor volume measurements in xenograft mouse models with FaDu/DDP cells. (**O**) Bioluminescence imaging of tumors. (**P**) Photographs of tumor tissues and tumor weight measurements. (**Q**) Immunohistochemical staining of xenograft tumors and positive-correlation statistics. Scale bars, 100 μm

Given the crucial role of PARP1 in DNA damage repair and its significant contribution to cisplatin resistance, we aimed to investigate whether the MRPL21-PARP1 axis regulated the sensitivity of HNSCC cells to cisplatin. Firstly, we measured the IC50 values of cisplatin in MRPL21-overexpressing and PARP1-knockdown cells, and found that the IC50 value of MRPL21-overexpressing cells increased, while the PARP1 knockdown rescue group exhibited no significant difference compared to the control group (Fig. 5A). We subsequently treated the cells with a median concentration of cisplatin and conducted CCK8, colony formation, and EdU assays to assess cellular viability and proliferation (Fig. 5A, B and E). Our results demonstrated that compared to the control group, cell viability was higher in the overexpressing group. TUNEL staining and flow cytometry further confirmed that the apoptotic rate was significantly lower in the overexpressing group. Conversely, these effects were reversed in the PARP1 knockdown rescue group (Fig. 5C–D). We also knocked down MRPL21 and overexpressed PARP1 in FaDu cells to comprehensively evaluate the regulatory effects of these two genes on cisplatin resistance in FaDu cells (Supplementary Fig. S4).

To more accurately simulate the conditions of cisplatin-resistant tumors and evaluate the role of MRPL21 in cisplatin resistance, we further validated our findings using the cisplatin-resistant FaDu cell line. We analyzed the expression levels of MRPL21 in the resistant cells and observed that it was significantly upregulated compared to the parental FaDu cells (Fig. 5F). Additionally, the resistant cells exhibited significantly reduced apoptosis following cisplatin treatment (Fig. 5G). Subsequently, we established stable cell lines with MRPL21 knockdown and with PARP1 overexpression in the resistant FaDu/DDP cells. The results indicated that MRPL21 knockdown decreased the IC50 value of cisplatin, whereas PARP1 overexpression reversed this effect (Fig. 5H). Furthermore, FaDu/DDP cells exhibited an enlarged cell size, increased intercellular spacing, and an irregular mesenchymal phenotype, which were mitigated upon MRPL21 knockdown (Fig. 5I). CCK8, colony formation, and EdU assays following cisplatin treatment demonstrated that cell viability was significantly reduced in the MRPL21 knockdown group. Flow cytometry and TUNEL staining further confirmed a marked increase in apoptosis in this group, and these effects were subsequently reversed upon PARP1 overexpression (Fig. 5J–M). To further validate these findings in vivo, we subcutaneously injected three groups of cells into nude mice and administered 5 mg/kg cisplatin via intraperitoneal injection every 3 days. In vivo imaging revealed that the cisplatin-resistant cells exhibited strong resistance to cisplatin-induced apoptosis and tumor growth inhibition. This resistance was significantly attenuated by MRPL21 knockdown, leading to a significant reduction in tumor growth rate, volume, and weight. Conversely, PARP1 overexpression restored the resistant phenotype (Fig. 5N–P). IHC analysis of the tumors showed downregulation of Ki67, MRPL21, and PARP1 in the knockdown group, while PARP1 and Ki67 expression levels in the PARP1-overexpressing rescue group were comparable to those in the control group (Fig. 5Q).

### MRPL21 regulates cisplatin response via promoting PARylation and inhibiting autophagy through the PI3K/AKT/mTOR signaling pathway

**Fig. 6:**
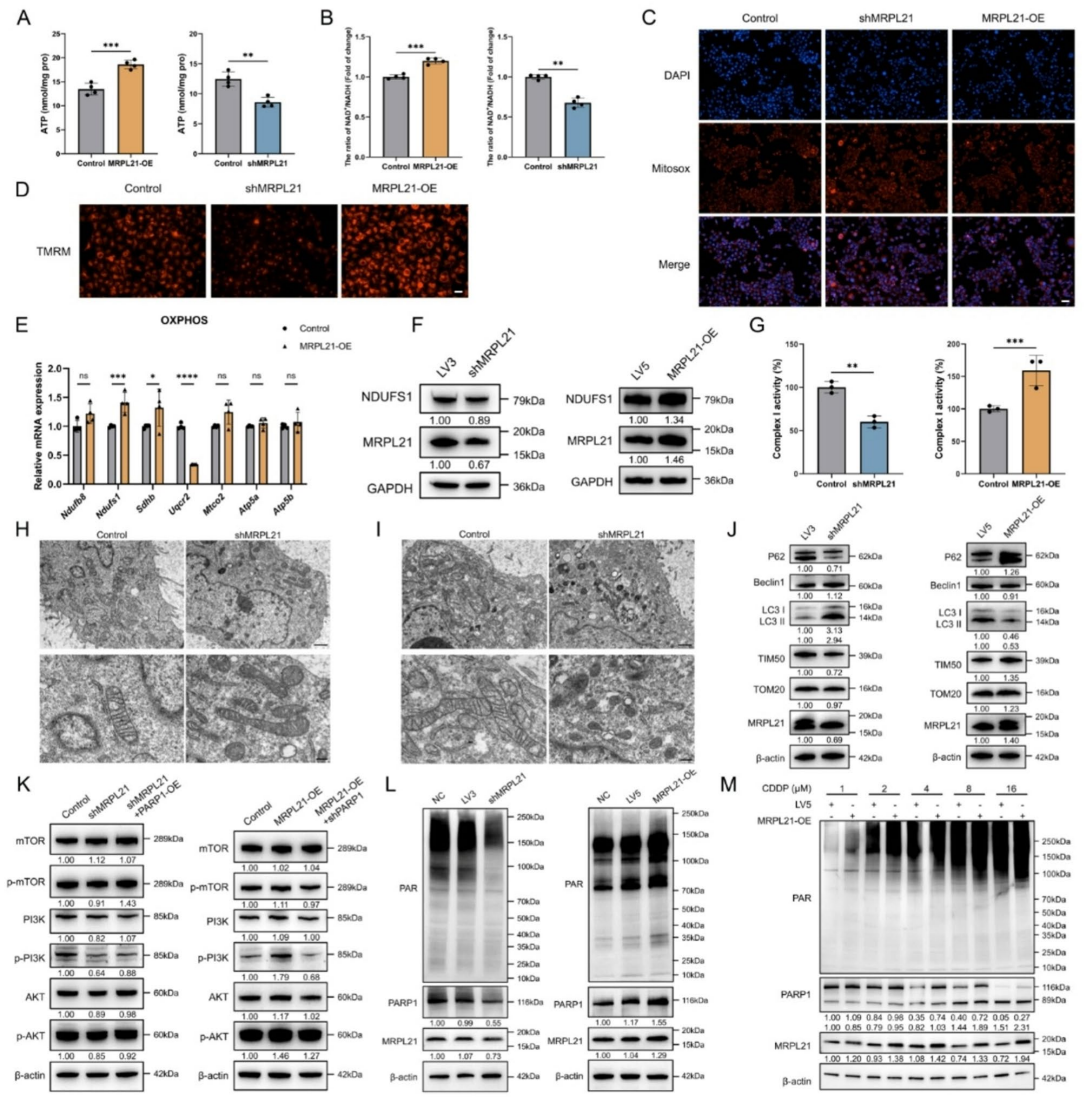
Effects of MRPL21 on mitochondrial function, PARylation and autophagy. (**A**) Bar graphs illustrating the levels of ATP in cells. (**B**) Bar graphs showing the ratios of NAD+/NADH in cells. (**C**) Fluorescence microscopic images of mitochondrial ROS in cells. Scale bars, 50 μm. (**D**) Fluorescence microscopic images demonstrating MMP. Scale bars, 50 μm. (**E**) Bar graph showing the relative mRNA expression levels of OXPHOS-related genes. (**F**) Western-blot analysis depicting the expression levels of NDUFS1. (**G**) Bar graph showing complex I activity in cells. (**H**) Transmission electron microscopic images showing mitochondrial structure and integrity. Scale bars, 1 μm (upper), 250 nm (below). (**I**) Transmission electron microscopic images showing mitochondrial structure and integrity after cisplatin treatment. Scale bars, 1 μm (upper), 250 nm (below). (**J**) Western blot analysis showing the expression levels of key proteins in mitochondrial autophagy. (**K**) Western blot analysis showing the expression levels of proteins in PI3K/AKT/mTOR pathway. (**L**) Western blot analysis showing the expression levels of PARylation. (**M**) Western blot analysis showing the change of PARylation level across different cisplatin concentration

Mitochondrial metabolism plays a critical role in tumor progression and cellular responses to chemotherapeutic agents, including cisplatin. Therefore, to elucidate the effects of MRPL21 on mitochondrial function, we evaluated cellular ATP production and NAD + levels. Results demonstrated that MRPL21-overexpressing cells had higher cellular ATP and NAD + levels. MRPL21 overexpression enhanced mitochondrial function, whereas its knockdown resulted in mitochondrial dysfunction (Fig. 6A–B). Specifically, MRPL21 knockdown increased reactive oxygen species (ROS) levels and reduced mitochondrial membrane potential (MMP), indicating that MRPL21 deficiency compromises normal mitochondrial activity. Conversely, high expression of MRPL21 in tumor cells promoted mitochondrial function (Fig. 6C–D). In order to further clarify the influence of MRPL21 overexpression on mitochondrial respiratory chain, we detected the expression of important coding genes of each subunit in the respiratory chain complexes, and found MRPL21 overexpression upregulated NDUFS1, a key subunit of electron transport chain complex I (Fig. 6E). Further analysis of potential MRPL21-binding proteins within mitochondria then led us to hypothesize that MRPL21 interacted with NDUFS1 (Supplementary Fig. S5A), and we discerned that NDUFS1 protein expression was positively correlated with MRPL21 levels (Fig. 6F). Both endogenous and exogenous Co-IP experiments confirmed the specific interaction between MRPL21 and NDUFS1 (Supplementary Fig. S5B and C), and immunofluorescence assays demonstrated their co-localization in the cytoplasm (Supplementary Fig. S5D). Functional studies revealed that MRPL21 knockdown decreased complex I activity, while its overexpression enhanced it (Fig. 6G).

To further observe the impact of MRPL21 expression on cell functions, we performed cryo-electron microscopy analysis on knockdown and control groups, and observed that compared to controls, mitochondria in the knockdown group appeared swollen with indistinct cristae, indicating impaired mitochondrial function (Fig. 6H). Given that apoptosis is a mitochondrion-dependent cell death process, we treated cells with low concentrations of cisplatin to observe MRPL21-related mitochondrial changes. We noted that mitochondria in the knockdown group lost their normal structure compared to controls, with the appearance of numerous autophagosomes within cells and a notable activation of the apoptotic process (Fig. 6I). We demonstrated that mitochondrial ROS levels were significantly increased after cisplatin treatment, and that MMP was markedly attenuated in the knockdown group (Supplementary Fig. S5E and F). Protein analysis revealed that after MRPL21 knockdown, the expression levels of LC3 II and Beclin1 were upregulated, while the expression level of P62 decreased, indicating a significant enhancement in autophagy levels. The levels of mitochondrial inner and outer membrane proteins TIM50 and TOM20 were reduced, suggesting that mitochondrial content and function were inhibited. Conversely, MRPL21 overexpression exerted the opposite effect (Fig. 6J). In order to further elucidate the specific mechanism by which MRPL21 influences autophagy, we performed additional analyses. It is widely recognized that the PI3K/AKT/MTOR pathway acts as a negative regulator of autophagy. The RNA-seq results revealed that the PI3K/Akt pathway was significantly enriched upon MRPL21 overexpression (Fig. 2G). Consistent with this, GSEA analysis of HALLMARK pathways demonstrated elevated MTORC1 signaling in the overexpression group (Supplementary Fig. S5G). Consequently, we examined the phosphorylation and activation status of PI3K/AKT/MTOR proteins in both the MRPL21 overexpression and knockdown cells, along with the corresponding PARP1 reverse cells. We observed that MRPL21 overexpression activated the pathway, whereas the PARP1-knockdown group exhibited an opposing phenotype. These results indicate that PARP1 expression modulates the phosphorylation levels of pathway proteins (Fig. 6K). Furthermore, PARP1 activity and PARylation can modify and regulate multiple pathways and downstream targets. Given that NAD + levels influence PARylation, we investigated whether MRPL21’s impact on mitochondria might affect PARylation in the cell. The data showed increased PARylation levels in the overexpression group and decreased levels in the knockdown group (Fig. 6L). Moreover, PARylation levels varied in accordance with the degree of apoptosis induced by cisplatin treatment (Fig. 6M). Collectively, these findings demonstrate that MRPL21 regulates PARylation levels and PI3K/AKT/MTOR pathway activity, thereby modulating cellular susceptibility to cisplatin via autophagy.

### Therapeutic targeting of MRPL21 in HNSCC

MRPL21 plays a critical role in the development, progression, and metastasis of HNSCC, regulating mitochondrial function and sensitivity to chemoradiotherapy. However, there is currently a lack of small-molecule inhibitors that target MRPL21. To address this gap, we aimed to develop a drug-delivery system capable of delivering MRPL21 siRNA into tumor cells, thereby effectively targeting the MRPL21 pathway and inhibiting tumor growth.

**Fig. 7:**
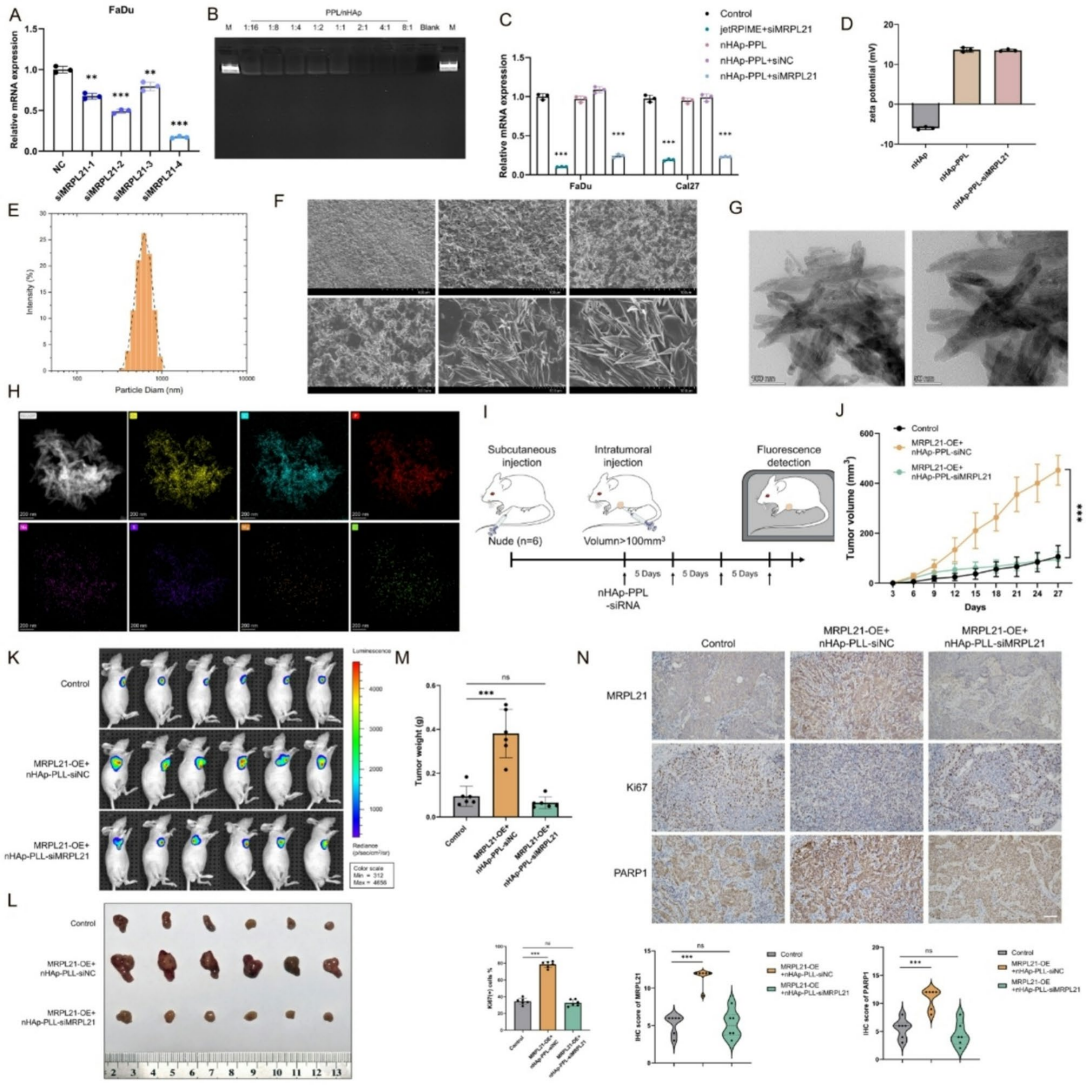
Targeting MRPL21 is an effective way to limit HNSCC progression. (**A**) Bar graph showing the efficiency of siMRPL21. (**B**) Gel-electrophoretic image showing the load capacity of different proportions. (**C**) Bar graph showing relative mRNA expression levels of MRPL21. (**D**) Zeta potential assay of nHAp-PLL-siMRPL21. (**E**) Dynamic light scattering size distribution of nHAp-PLL-siMRPL21. (**F-H**) TEM and SEM images of nHAp-PLL-siMRPL21. (**I**) Schematic diagram of the experimental design for the subcutaneous and intratumoral injections of nude mice. (**J**) Line graph showing tumor volume measurements over time in different treatment groups. (**K**) Photographs of tumor tissues. (**L**) Bioluminescence imaging of tumor growth in nude mice. (**M**) Bar graph detailing tumor weights. (**N**) Immunohistochemical staining of tumor sections showing the expression levels of xenograft tumors and positive-correlation statistics. Scale bars, 100 μm

Hydroxyapatite (HAp) is a widely utilized material in nanodrug synthesis, and drawing on previous research we coated nano-sized hydroxyapatite (nHAp) with poly-L-lysine (PLL) to synthesize submicron-sized drug-delivery complexes capable of transporting siRNA into tumor cells. Initially, we identified the most effective MRPL21 siRNA sequence (Fig. 7A). and then optimized the PLL/nHAp mass ratio for efficient siRNA encapsulation and determined that a PLL/nHAp ratio of 4:1 yielded the highest loading efficiency (Fig. 7B). The gene-silencing efficiency of the delivery system was validated by using qRT-PCR, demonstrating an efficiency comparable to that achieved with jetPRIME transfection reagents (Fig. 7C). We measured the zeta potential of the complexes and confirmed that nHAp exhibited a zeta potential of − 6.007 ± 0.3602 mV. Following PLL coating, the zeta potential shifted to 13.63 ± 0.5508 mV, resulting in a positively charged complex capable of electrostatically interacting with negatively charged siRNA for efficient transport and controlled release. Upon siRNA loading, the zeta potential slightly decreased to 13.50 ± 0.2646 mV (Fig. 7D). Dynamic light scattering (DLS) analysis revealed that the particle size of the complexes was approximately 867 nm, confirming the submicron size of the synthesized particles (Fig. 7E). Scanning electron microscopy (SEM) and transmission electron microscopy (TEM) assessments revealed that the particles possessed a rod-like and needle-like morphology with excellent dispersity. Energy-dispersive X-ray spectroscopy (EDS) analysis further characterized the elemental composition of the complexes, and identified elements such as Ca, O, P, Na, S, Mg, and Cl (Fig. 7F–H).

To validate the efficiency of nHAp-PLL-siMRL21 in vivo, we performed experiments in nude mice using subcutaneous tumor xenografts. When tumor volumes reached approximately 100 mm^3^, intratumoral injections of the complexes were initiated at a dose of 5 mg/kg that carrying 1 mg/kg of siRNA, and administered the dose every five days for a total of four injections (Fig. 7I). In vivo imaging showed that tumor proliferation was significantly suppressed in mice treated with the nanocompound compared to the overexpression group, with tumor volume and weight significantly reduced to levels comparable to those of the blank-control group (Fig. 7J–M). IHC analysis of tumor tissues further revealed significant downregulation of MRPL21, Ki67, and PARP1 in the drug-treated group (Fig. 7N).

### MRPL21-PARP1 axis promotes cisplatin resistance in HNSCC by PI3K/AKT/mTOR-autophagy pathway, and is targetable by nanotherapeutic SiRNA delivery

**Fig. 8:**
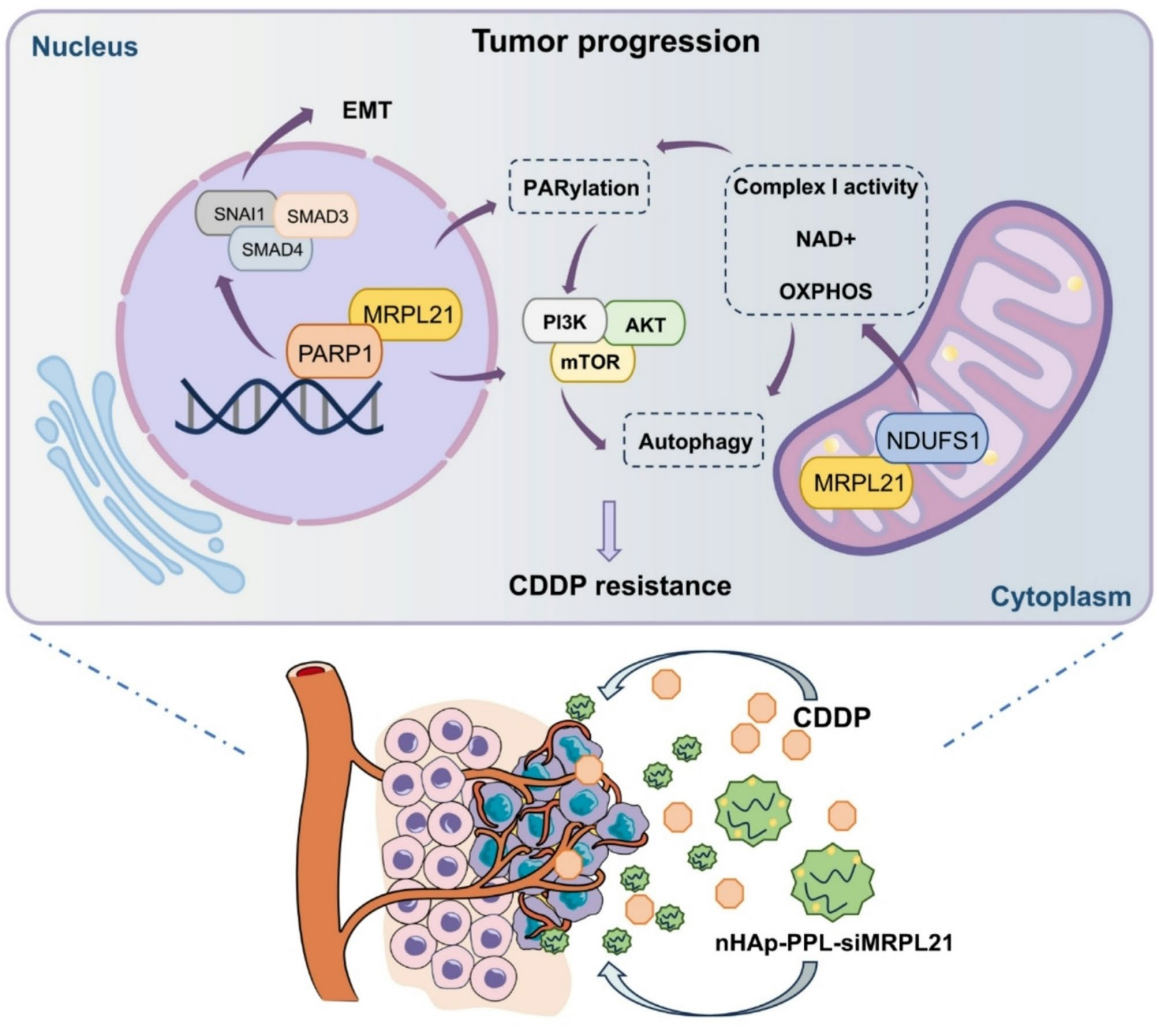
Diagram of tumorigenic mechanism of MRPL21 MRPL21 regulates tumorigenesis and development in head and neck squamous cell carcinoma (HNSCC) through interactions with PARP1 to regulate EMT progress and CDDP resistance. MRPL21 enhances mitochondrial function, regulates PARylation in cells, and further influences autophagy through PI3K/AKT/mTOR pathway, thereby affecting cell sensitivity to cisplatin therapy. Nanomedical drug delivery systems targeting MRPL21 with siRNAs can be used as sensitizers for CDDP therapy and by regulating PI3K/AKT/mTOR-regulated autophagy, thereby overcoming cisplatin resistance to HNSCC

Through single-cell sequencing, we identified a subpopulation of cancer cells in HNSCC tumor tissues and metastatic lymph node tissues that express the potential oncogene MRPL21. MRPL21 exhibits tumor-regulatory functions independent of its canonical mitochondrial ribosomal role by interacting with PARP1 in the nucleus, thereby modulating tumor proliferation, migration and EMT processes, as well as cellular sensitivity to chemotherapeutic agents such as cisplatin. Overexpression of MRPL21 enhances the activity of mitochondrial respiratory chain complex I via interaction with NDUFS1, thereby promoting OXPHOS and NAD + level. Overexpression of MRPL21 significantly promotes PARP1 activity and PARylation, MRPL21-PARP1 axis subsequently activates the PI3K/AKT/mTOR signaling pathway to suppress autophagy. This process affects drug-induced apoptosis and promotes cisplatin resistance in HNSCC. Our study demonstrates that PLL-modified nHAp can effectively deliver MRPL21 siRNA to inhibit tumor growth in vivo, underscoring MRPL21 as a promising therapeutic target and chemosensitizer for head and neck cancer (Fig. 8).

## Discussion

The prognosis of patients with HNSCC is generally poor [32], with a 5-year survival rate ranging from 40 to 50%; and up to 30% of HNSCC patients experience cancer recurrence and treatment failure [33]. Previous studies showed that the prognosis of hypopharyngeal cancer patients was associated with factors such as age, TNM staging, site of origin, whether surgical treatment was performed on the primary tumor, and the site of tumor metastasis [34]. Therefore, an increasing number of investigators currently focus on evaluating biomarkers and gene-expression profiles that can predict patient prognosis and treatment sensitivity, to develop personalized treatment plans and predict disease progression and response to treatment. Thus, the search for new therapeutic targets and chemosensitizers for HNSCC is paramount.

With the rapid advancements in single-cell sequencing technology, its pivotal role in tumor research has been further enhanced. This technique enables the quantification of variant copy numbers, thereby uncovering genomic heterogeneity [35], effectively stratifies cell populations and identifies rare cells [36], and provides a theoretical basis for the early screening of cancer patients and the development of personalized clinical treatment strategies [37]. Single-cell sequencing has significant implications for the development of molecularly targeted therapeutic strategies, the prevention of chemotherapeutic resistance, and tumor recurrence [38]. In our preliminary study, single-cell RNA sequencing analysis was conducted on normal adjacent tissues, tumor tissues, and metastatic lymph node tissues from five patients with HNSCC. This analysis facilitated a deeper understanding of the heterogeneity of NATs in HSCC [39] and provided a comprehensive characterization and investigation of the immune microenvironment in HSCC [40]; this process then facilitated a more precise analysis of the potential roles of different cellular subpopulations and immune infiltration during the development and progression of HSCC. Based on previous sequencing data, we identified tumor cell subpopulations that were specifically present in tumor tissues and metastatic lymph nodes. Based on highly expressed genes, we further subdivided the tumor cells into six distinct subpopulations, and subsequent functional enrichment analysis of these subpopulations revealed a subpopulation that is associated with malignant characteristics such as cell cycle progression. We analyzed both highly expressed genes in this subpopulation and in tumor and metastatic tissues, and identified the potentially oncogenic gene MRPL21. Validation using online databases and confirmation in HSCC tissues collected by our research group demonstrated that MRPL21 was highly expressed in head and neck tumors and was significantly associated with poor patient prognosis and reduced survival.

MRPL21 is a gene that encodes mitochondrial ribosomal protein L21, a component of the mitochondrial ribosome that plays an essential role in mitochondrial protein synthesis. Altered expression of mitochondrial ribosomal proteins (MRPs) has been observed in various cancer types and is associated with clinical features of certain cancers [41]. Specifically, the downregulation of MRPL13, a key regulator of mitochondrial ribosome function, impairs oxidative phosphorylation (OXPHOS) and contributes to the invasive activity of hepatocellular carcinoma (HCC) [42]. Additionally, MRPL33 promotes colorectal cancer cell proliferation and inhibits apoptosis [43, 44], while its reduced expression induces mitochondrial dysfunction and enhances tumor cell apoptosis [45]. The SIRT1/MRPS4 axis is involved in metabolic reprogramming, facilitates tumor progression in HCC and plays a critical role in maintaining the stemness of liver cancer stem cells (CSCs) and participating in tumor progression [46]. In this study, we demonstrated that MRPL21 exhibits significant oncogenic properties in HNSCC, promoting tumor cell proliferation, migration, and invasion. Knockdown of MRPL21 attenuates the malignant phenotype of tumor cells, whereas its overexpression is closely linked to multiple tumor-related signaling pathways, regulating critical processes such as the G2M checkpoint and cell cycle progression, as well as being associated with key mutated genes in cancer.

Mitochondrial OXPHOS plays a critical role in regulating tumor metastasis [47]. Various mitochondrial ribosomal proteins regulate the metastatic and invasive phenotypes of tumors by modulating intracellular ROS and OXPHOS levels [48, 49]. Consequently, we examined the impact of MRPL21 expression on the EMT phenotype. Our results demonstrated that overexpression of MRPL21 downregulated epithelial markers while upregulating mesenchymal markers in tumor cells, suggesting that MRPL21 promotes EMT in these cells. Furthermore, knockdown of MRPL21 restored the epithelial phenotype in cisplatin-resistant cell lines. This regulation of the EMT by MRPL21 is now known to be achieved through the modulation of molecules such as ZEB1, Smad4, and p65 by PARP1.

PARP1 is a crucial member of the PARP family, which modifies targets through ADP-ribosylation and plays key roles in a variety of biological processes. PARP1 exhibits distinct responses to different types of DNA damage, being rapidly recruited to the damaged site and activated with significant variations in its activity under various stimuli [50, 51]. It is also involved in a wide range of cellular activities, including DNA damage repair, DNA replication, transcriptional regulation, ribosomal biogenesis, and programmed cell death [52]. Overexpression of PARP1 has been observed in various cancers, highlighting its clinical potential as a therapeutic target for human malignancies. In the current study, we first investigated the potential extramitochondrial functions of MRPL21, and our results revealed a potential interaction between MRPL21 and PARP1. MRPL21 promotes tumor proliferation and metastasis by regulating PARP1 expression, and knockdown of PARP1 reverses the oncogenic effects of MRPL21. Additionally, studies have shown that MRPs are not only involved in mitochondrial energy supply and OXPHOS but also in the regulation of cellular states such as the induction of apoptosis [53]. PARP1 activation is acknowledged to be an early molecular marker of programmed cell death. Herein, we demonstrated that MRPL21 regulates PARP1 to inhibit the apoptotic response of HNSCC cells to chemotherapeutic drugs such as cisplatin, and the knockdown of the PARP1 gene can reverse this drug resistance. Subsequent validation using the cisplatin-resistant cell line further supported these findings.

Given the critical roles of PARP1 in cancer and DNA damage repair, PARP1 has emerged as a promising target in cancer therapy. Although several PARP1 inhibitors have been developed and approved for clinical use, the emergence of drug resistance and adverse effects in patients presents significant obstacles to their efficacy [54, 55]. To address these challenges, an increasing number of studies have focused on identifying novel PARP1 inhibitors, developing dual-target PARP1 inhibitors [56, 57], and discovering PARP1-inhibitor sensitizers [58] for combination therapies. In this study, we identified that the mitochondrial ribosomal protein MRPL21 potentially regulates PARP1 expression. The interaction between MRPL21 and PARP1 triggers apoptotic responses in cells, indicating that MRPL21 inhibitors may serve as potential sensitizers for PARP1.

Mitochondria can regulate the onset and development of tumors, and mitochondrial ribosomes are responsible for assisting in the synthesis of mitochondrial proteins and are thus essential for OXPHOS and ATP generation [59]. Based on NAD+, PARP transfers ADP-ribose groups to itself or other proteins to form polyADP-ribose (PAR) chains. PARylation modifications recruit multiple DNA repair proteins to the site of damage and regulate DNA damage repair as well as mitochondrial transcriptional regulation processes [27, 60]. Studies have shown that an increase in levels of NAD + activates PARP1, contributing to the widespread occurrence of PARylation modifications [27]. PARP1 and PARylation are involved in regulating the activation of various pathways, such as inhibitors of polyADP-ribosylation significantly inhibit the phosphorylation activation of AKT [61] and activate and regulate mTOR signal transduction [62]. PARP1 has been shown to promote EGFR-TKI resistance in non-small cell lung cancer through the PI3K/AKT pathway [63]. In this study, we found that MRPL21 interacted with NDUFS1 and promoted mitochondrial OXPHOS function and NAD + level enhancing energy production and proliferation in head and neck tumors.

Under normal circumstances, autophagy within cells is at a low base level, but overactivation of autophagy can also lead to cell death. The relationship between autophagy and apoptosis is also very complex, autophagy can inhibit, promote, or accompany apoptosis. Activation or inhibition of autophagy plays a crucial role in tumorigenesis, and it can exert powerful anti-tumor activity by inducing autophagy to occur in tumor cells [64]. The interaction between apoptosis and autophagy in cisplatin chemotherapy presents different states in different tumors [65, 66]. The PI3K/AKT/mTOR pathway is known to inhibit autophagy [67] and the PI3K/Akt pathway has been shown to participate in cisplatin-induced apoptosis and influence cisplatin resistance [68]. Studies shown that PI3K/AKT/mTOR/autophagic axis can promote intrahepatic cholangiocarcinoma progression and desensitizes cisplatin treatment [69]. In this study, we found that MRPL21 overexpression can significantly increase the PARylation level of cells and reduce the level of intracellular autophagy after cisplatin treatment. We demonstrated that inhibiting MRPL21 expression induced mitochondrial damage that led to increased autophagy within cells. This mitochondrial damage was exacerbated upon cisplatin treatment, with a significant increase in autophagosomes and a marked diminution in the content of mitochondrial inner and outer membrane proteins, ultimately inducing apoptosis associated with mitochondrial dysfunction. This phenotype may be related to the significantly enhanced PARylation and activation of PI3K/AKT/MTOR pathway after MRPL21 overexpression, resulting in inhibition of autophagy and cisplatin resistance.

Targeted drug delivery via nanocomplexes has long been a focal point in pharmacologic research endeavors. Nano-hydroxyapatite (nHAp) is a novel inorganic ceramic material with high chemical reactivity, which enhances its bioactivity. Its increased surface area facilitates cell attachment and growth. The nHAp also exhibits favorable biocompatibility and biodegradability and has been widely applied in various types of tissue repair [70]. Studies have demonstrated that surface modifications of nHAp can alter its surface potential and properties, thereby enhancing its efficacy as a drug carrier [71]. Cationic peptides (such as PLL) have good biocompatibility and low toxicity and are commonly used for modification. PLL is greatly capable of protecting DNA against enzymatic degradation in biological environments by forming a polyplex, leading to an efficient cellular internalisation as a gene carrier [72, 73]. In this study, we employed PLL to modify nHAp [74], explored its optimal loading efficiency and pH, and validated its transfection efficiency. Submicron drug-delivery systems have revealed significant advantages in the treatment of various tumors. Cisplatin combined with siMPRL21 nanodrug delivery system may benefit patients with cisplatin-resistant head and neck cancer.

## Conclusions

In this study, we utilized single-cell RNA sequencing (scRNA-seq) and tissue validation to confirm that MRPL21 was highly expressed and correlated with poor patient prognosis in HNSCC. MRPL21 promoted OXPHOS, modulated PARP1 activity, and influenced the downstream PI3K-AKT-mTOR signaling pathway. These regulatory effects collectively governed chemotherapy-induced autophagy, thereby affecting tumor proliferation and chemoresistance. Moreover, nanodrugs targeting MRPL21 exhibited substantial antitumor efficacy in in vivo models. Collectively, these findings underscore the dual potential of MRPL21 as a therapeutic target and chemotherapy sensitizer for HNSCC.

## Electronic supplementary material

Below is the link to the electronic supplementary material.

Supplementary Material 1

## Data Availability

No datasets were generated or analysed during the current study.

## Abbreviations

HNSCC: Head and neck squamous cell carcinoma
MRPs: Mitochondrial ribosomal proteins
MRPL21: Mitochondrial Ribosomal Protein L21
OXPHOS: Oxidative phosphorylation
HSCC: Hypopharyngeal squamous cell carcinoma
scRNA analysis: Single-cell RNA analysis
PARP1: Poly ADP-ribose Polymerase 1
PARylation: Poly ADP-ribosylation
EMT: Epithelial-mesenchymal transition
NDUFS1: NADH: Ubiquinone Oxidoreductase Core Subunit S1
OD: Optical density
EdU: 5-ethynyl-2’-deoxyuridine
Co-IP: Co-immunoprecipitation
LC‒MS/MS: Liquid Chromatograph Mass Spectrometer
IF: Immunofluorescence
IHC: Immunohistochemical
HE: Hematoxylin–eosin
Δψm: Mitochondrial membrane potential
TMRE: Tetramethylrhodamine ethyl este
ROS: Reactive oxygen species
DLS: Dynamic light scattering
TEM: Transmission electron microscop
FE-SEM: Field-emission scanning electron microscopy
EDS: Energy-dispersive spectroscopy
