# Supplementary material for: MRPL21-PARP1 axis promotes cisplatin resistance in head and neck squamous cell carcinoma by inhibiting autophagy through the PI3K/AKT/mTOR signaling pathway
Source: J Exp Clin Cancer Res. 2025 Jul 26;44:221. doi: 10.1186/s13046-025-03482-9 (PMC12297673; doi:10.1186/s13046-025-03482-9)

Fig. 1L

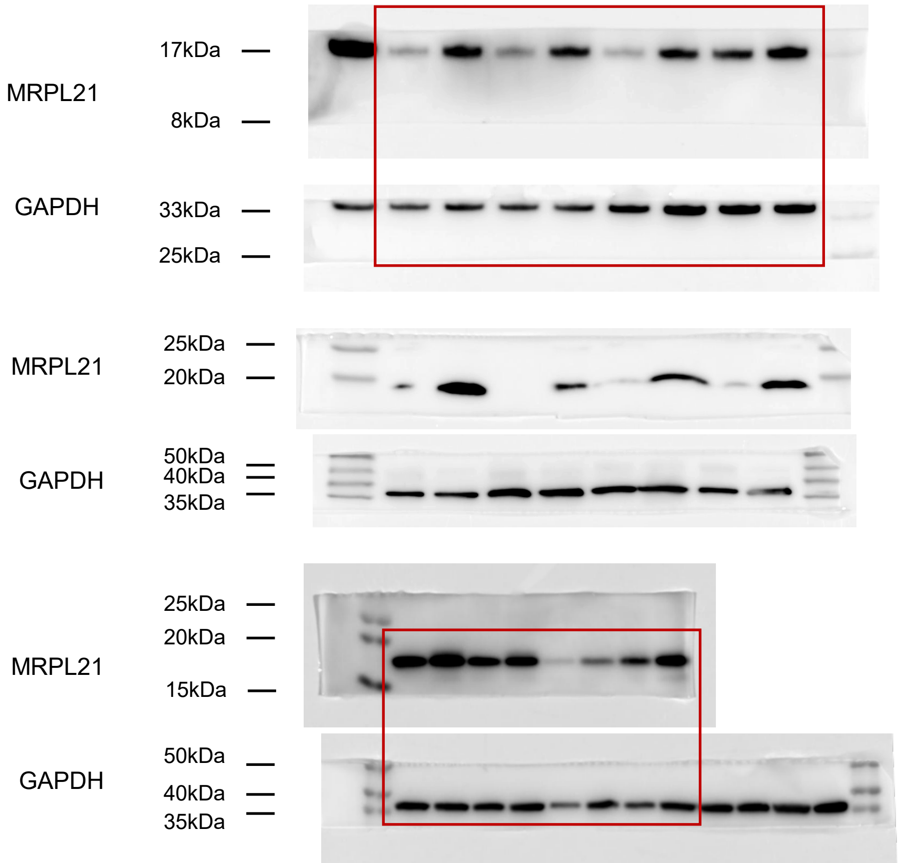

Fig. 2A

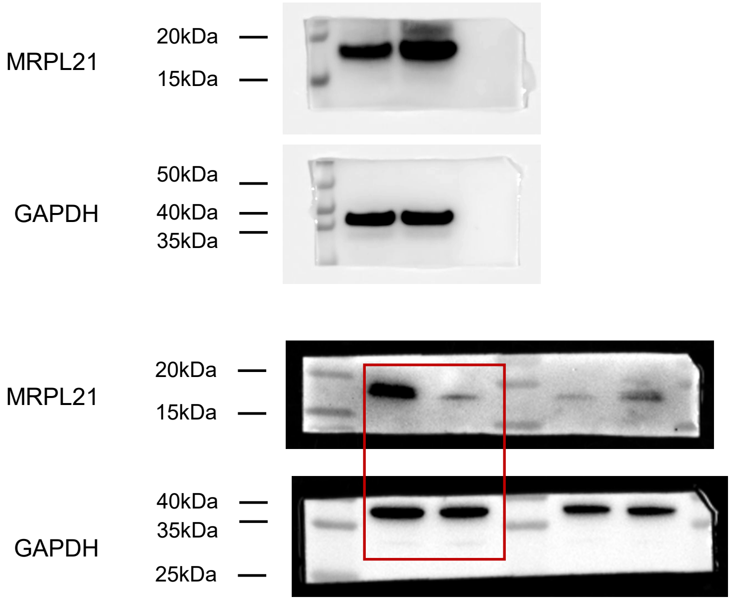

Fig. 2I Left

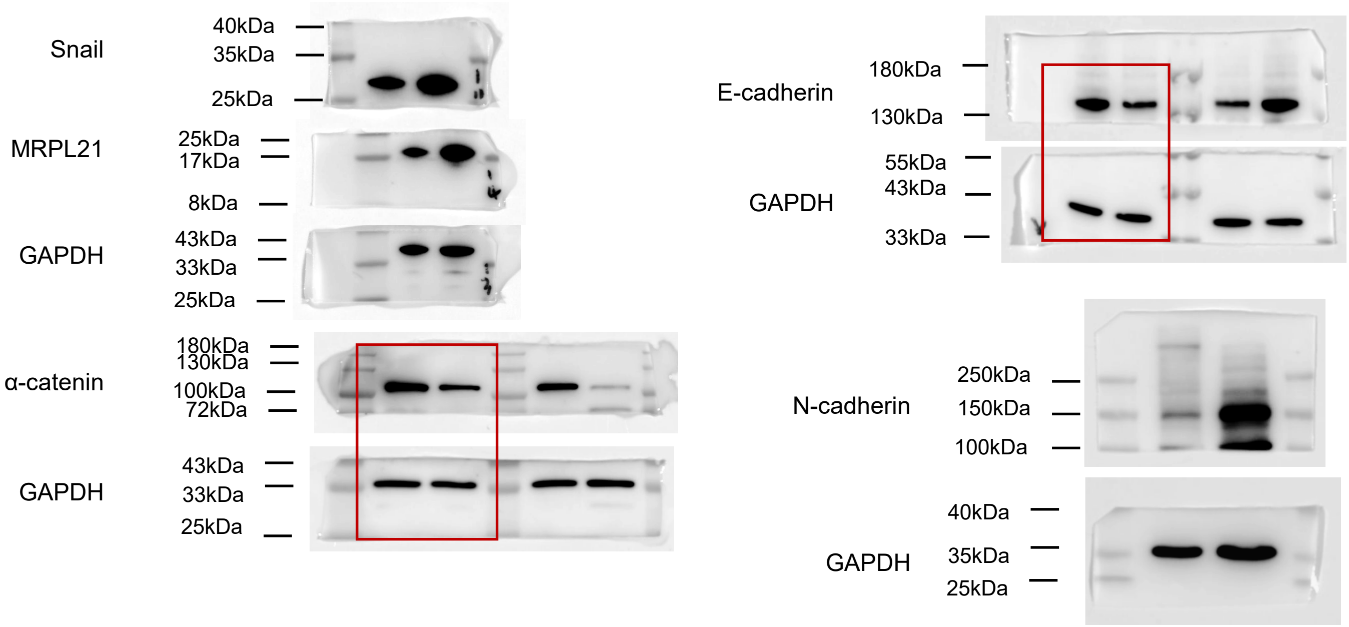

Fig. 2I Right

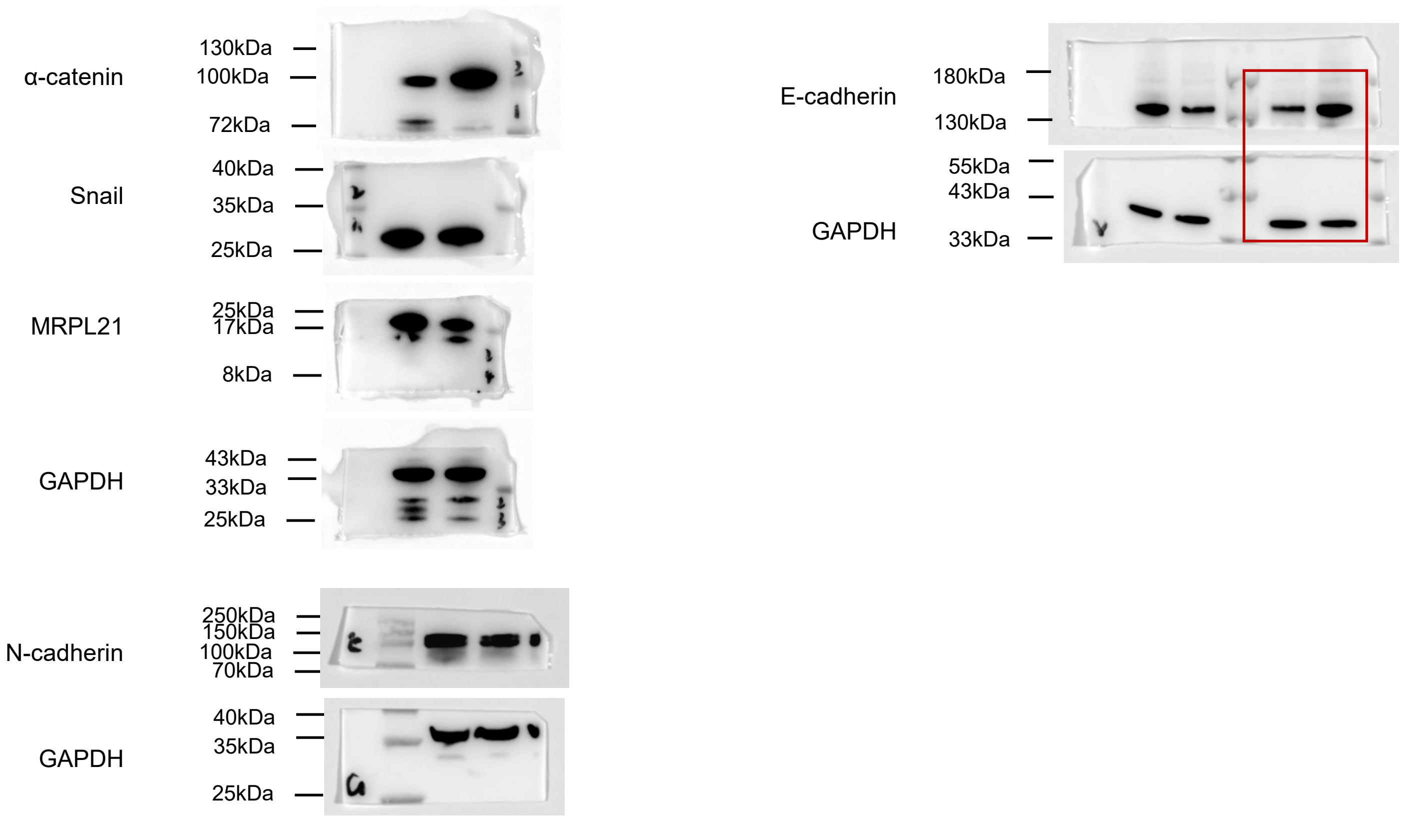

Fig. 3B

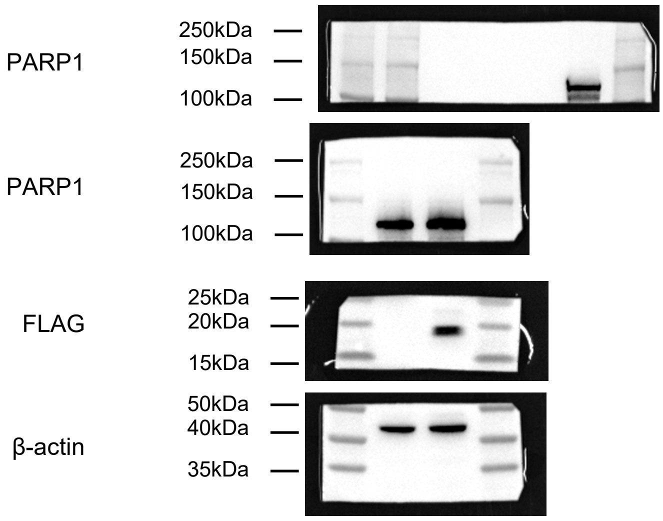

Fig. 3C

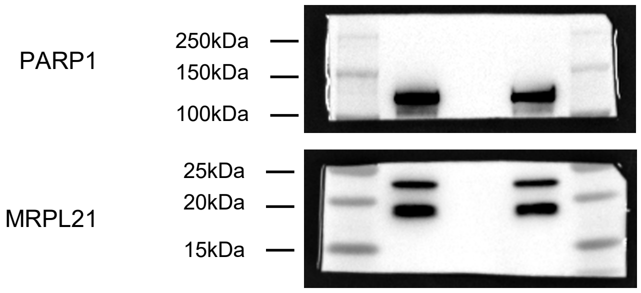

Fig. 3D

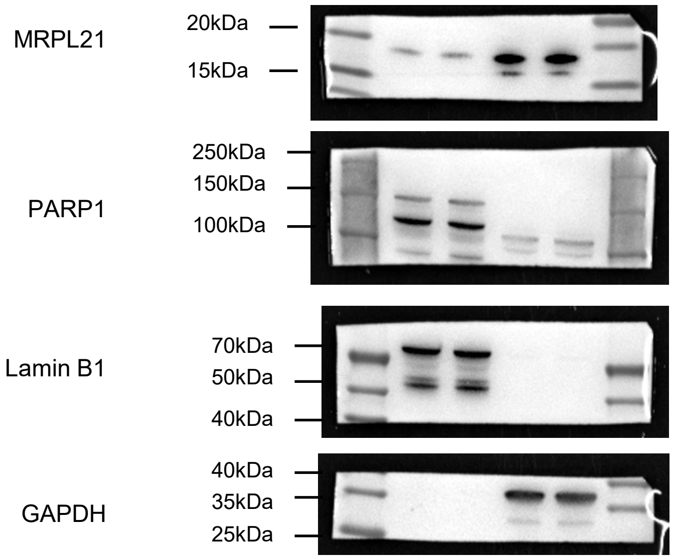

Fig. 3F

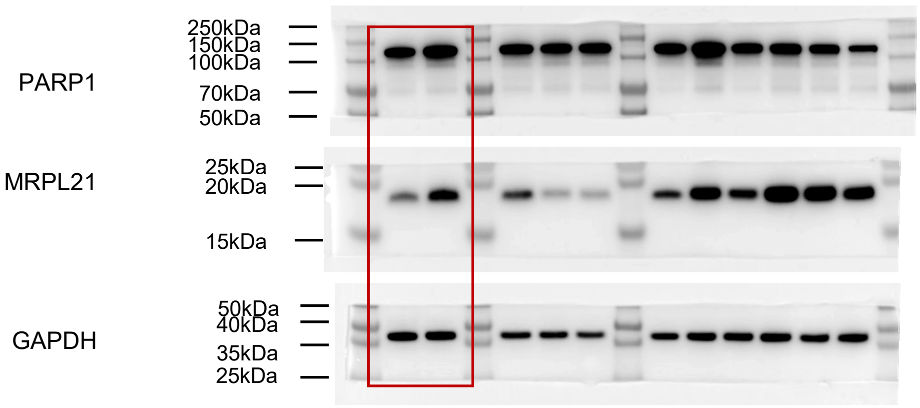

Fig. 3G

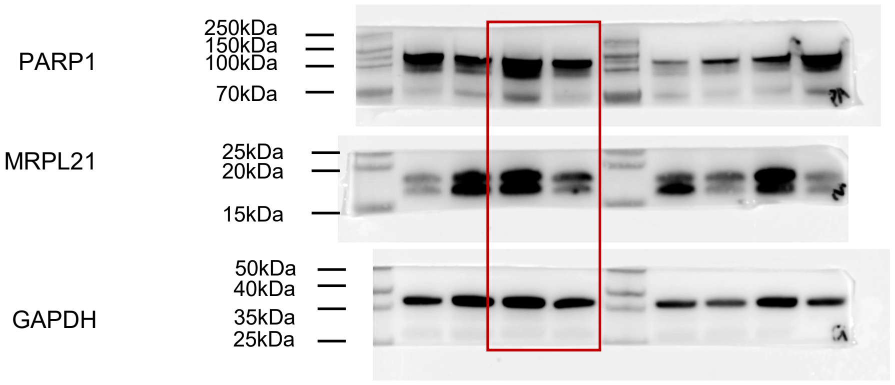

Fig. 3H

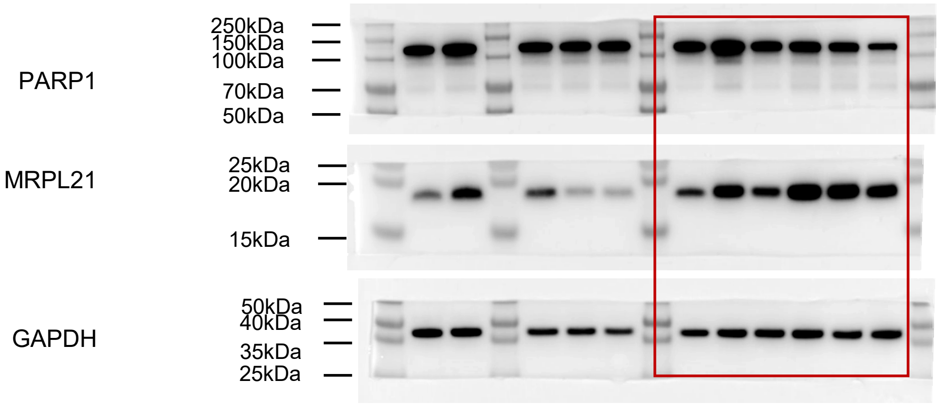

Fig. 3P

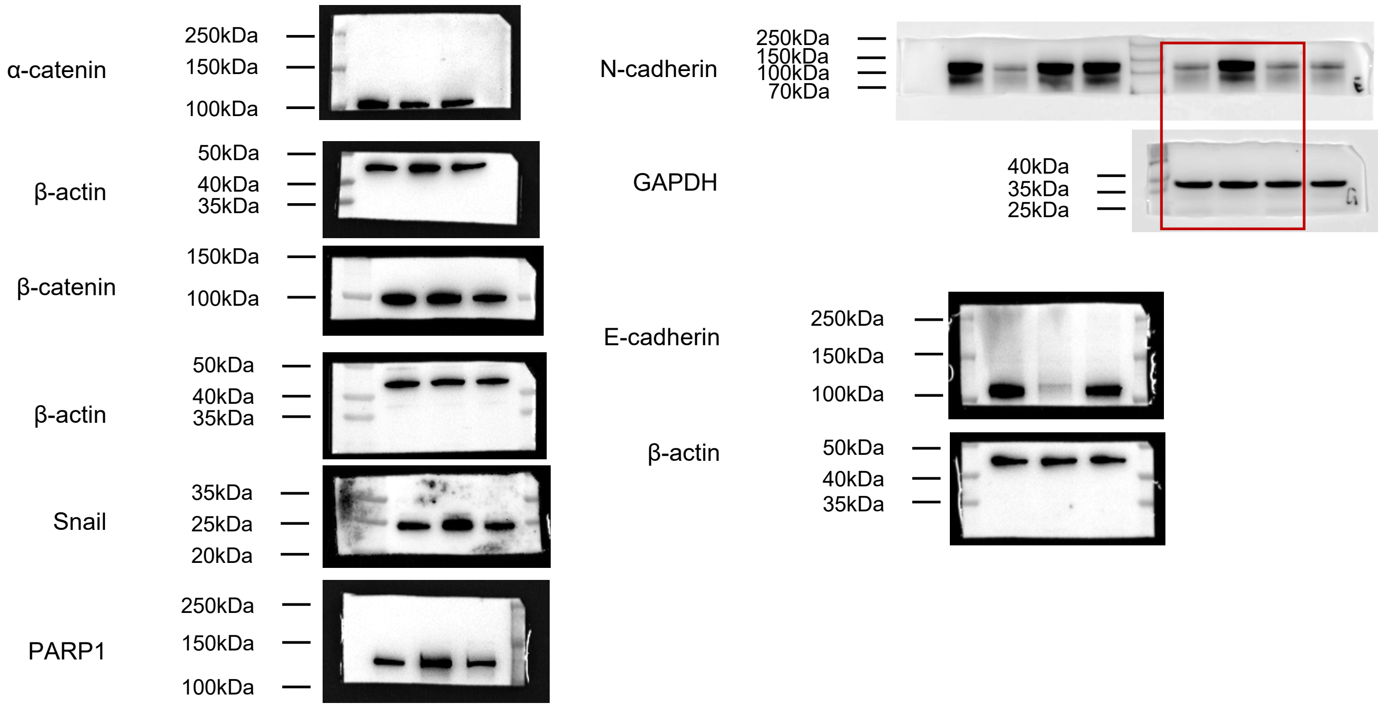

Fig. 3Q

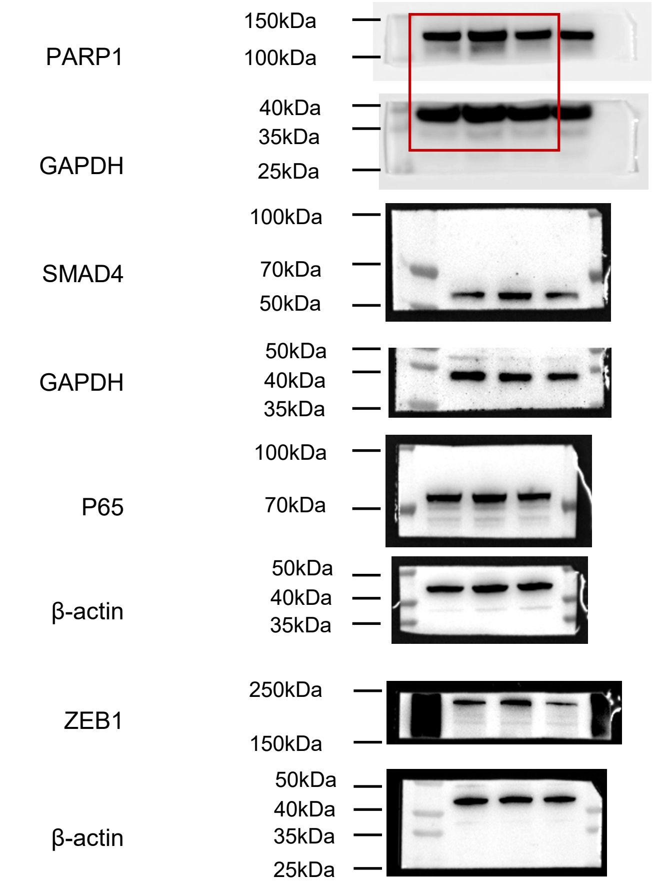
PARP1
150kDa
100kDa
GAPDH
40kDa
35kDa
25kDa
SMAD4
100kDa
70kDa
50kDa
GAPDH
50kDa
40kDa
35kDa
P65
100kDa
70kDa
β-actin
50kDa
40kDa
35kDa
ZEB1
250kDa
150kDa
β-actin
50kDa
40kDa
35kDa
25kDa

Fig. 4F

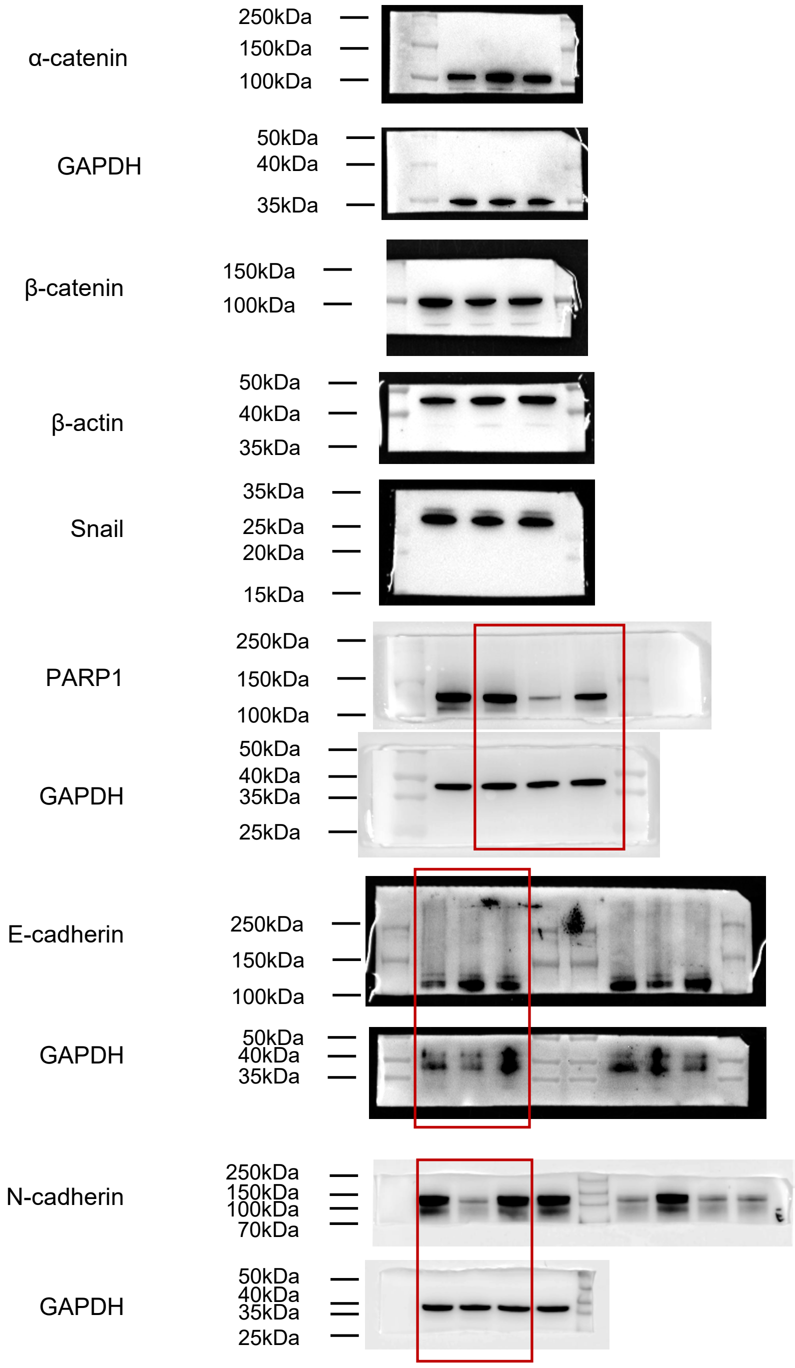
α-catenin
250kDa
150kDa
100kDa
GAPDH
50kDa
40kDa
35kDa
β-catenin
150kDa
100kDa
β-actin
50kDa
40kDa
35kDa
Snail
35kDa
25kDa
20kDa
15kDa
PARP1
250kDa
150kDa
100kDa
GAPDH
50kDa
40kDa
35kDa
25kDa
E-cadherin
250kDa
150kDa
100kDa
GAPDH
50kDa
40kDa
35kDa
N-cadherin
250kDa
150kDa
100kDa
70kDa
GAPDH
50kDa
40kDa
35kDa
25kDa

Fig. 4G

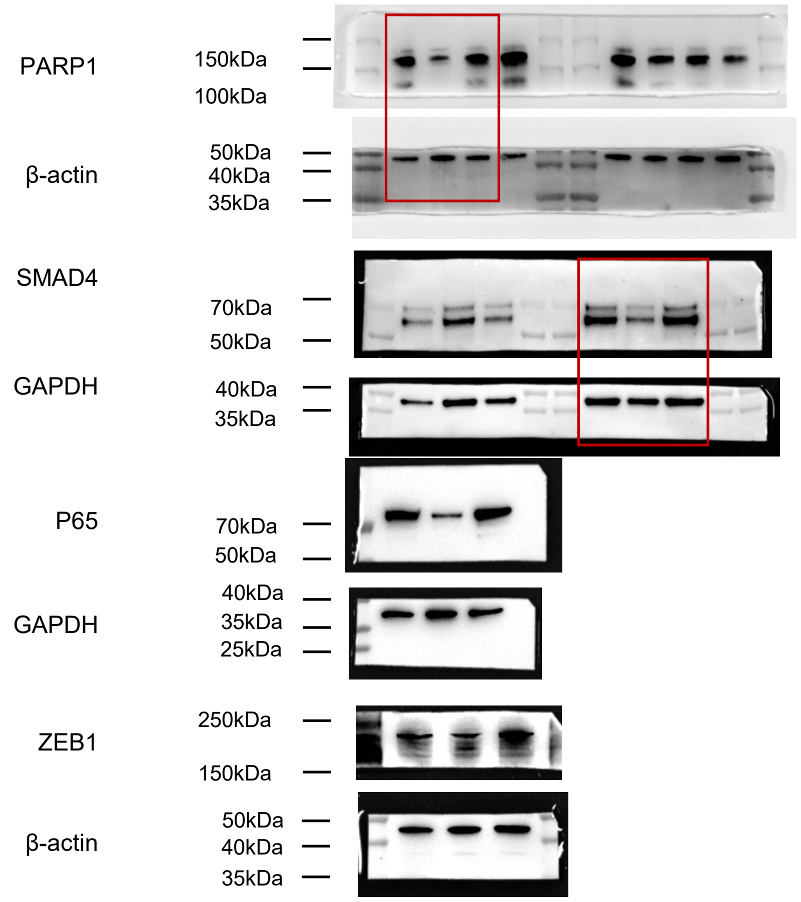

Fig. 5F

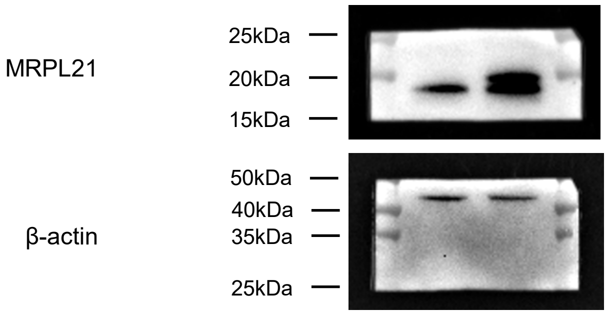

Fig. 6F

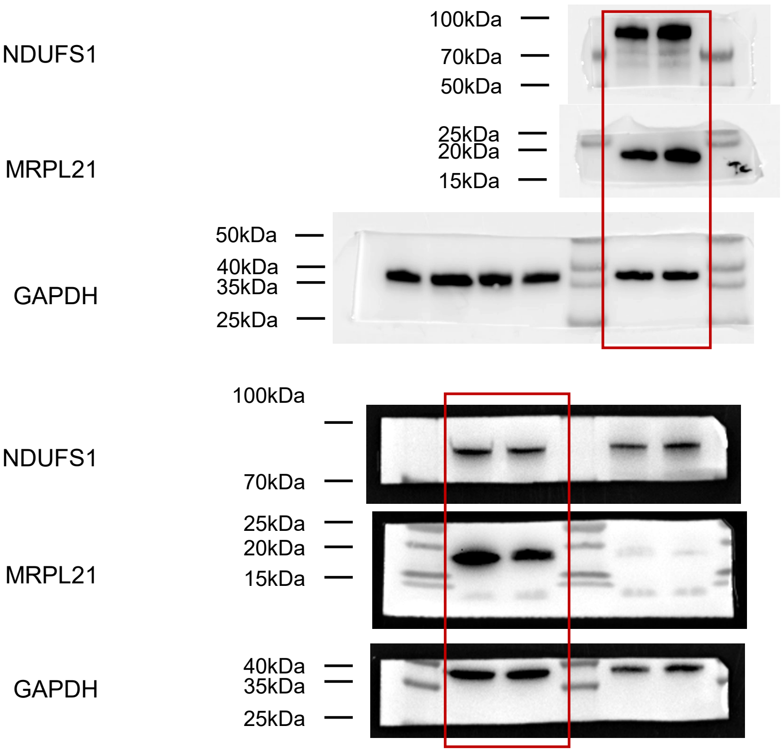

Fig. 6J Left

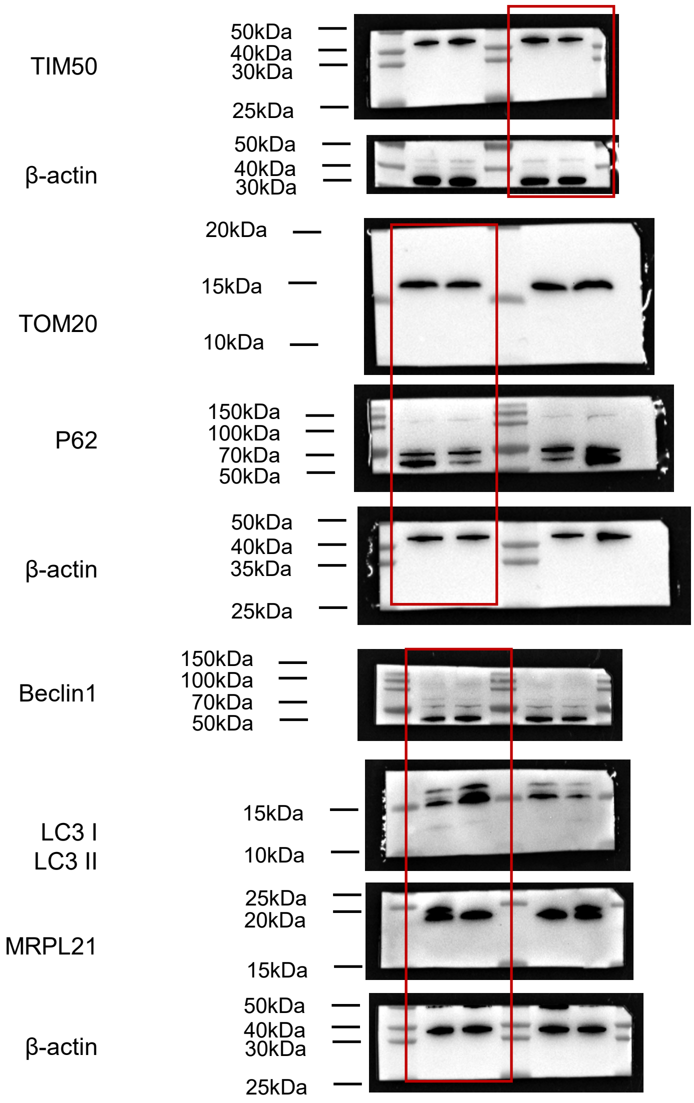

Fig. 6J Right

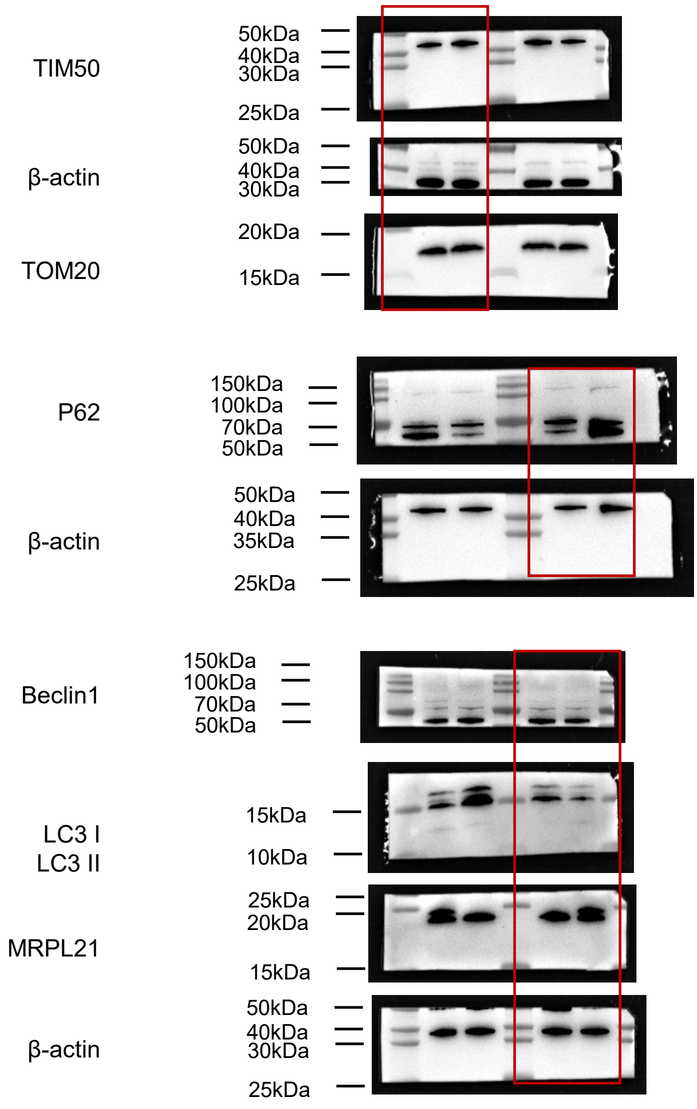

Fig. 6K Left

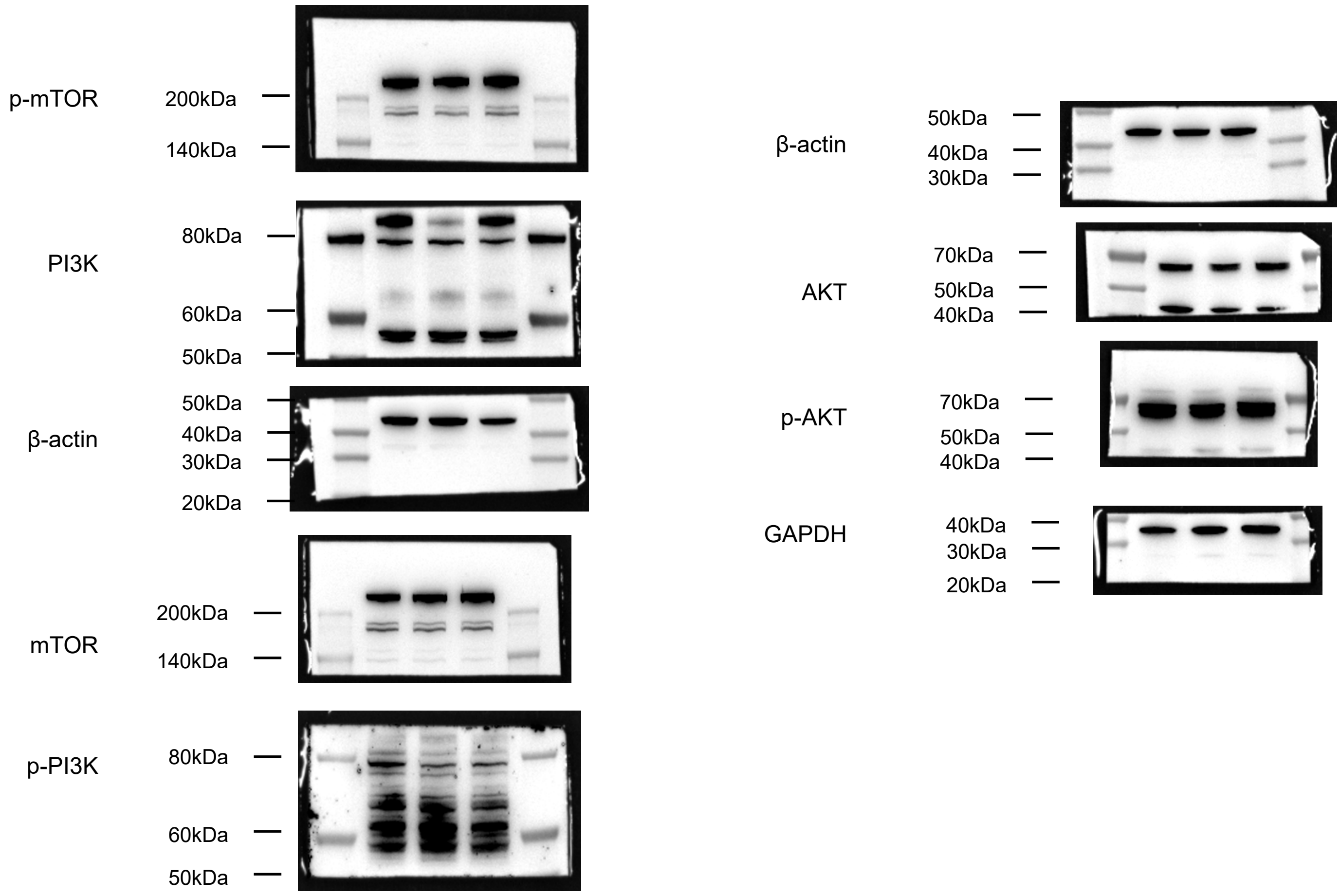

Fig. 6K Right

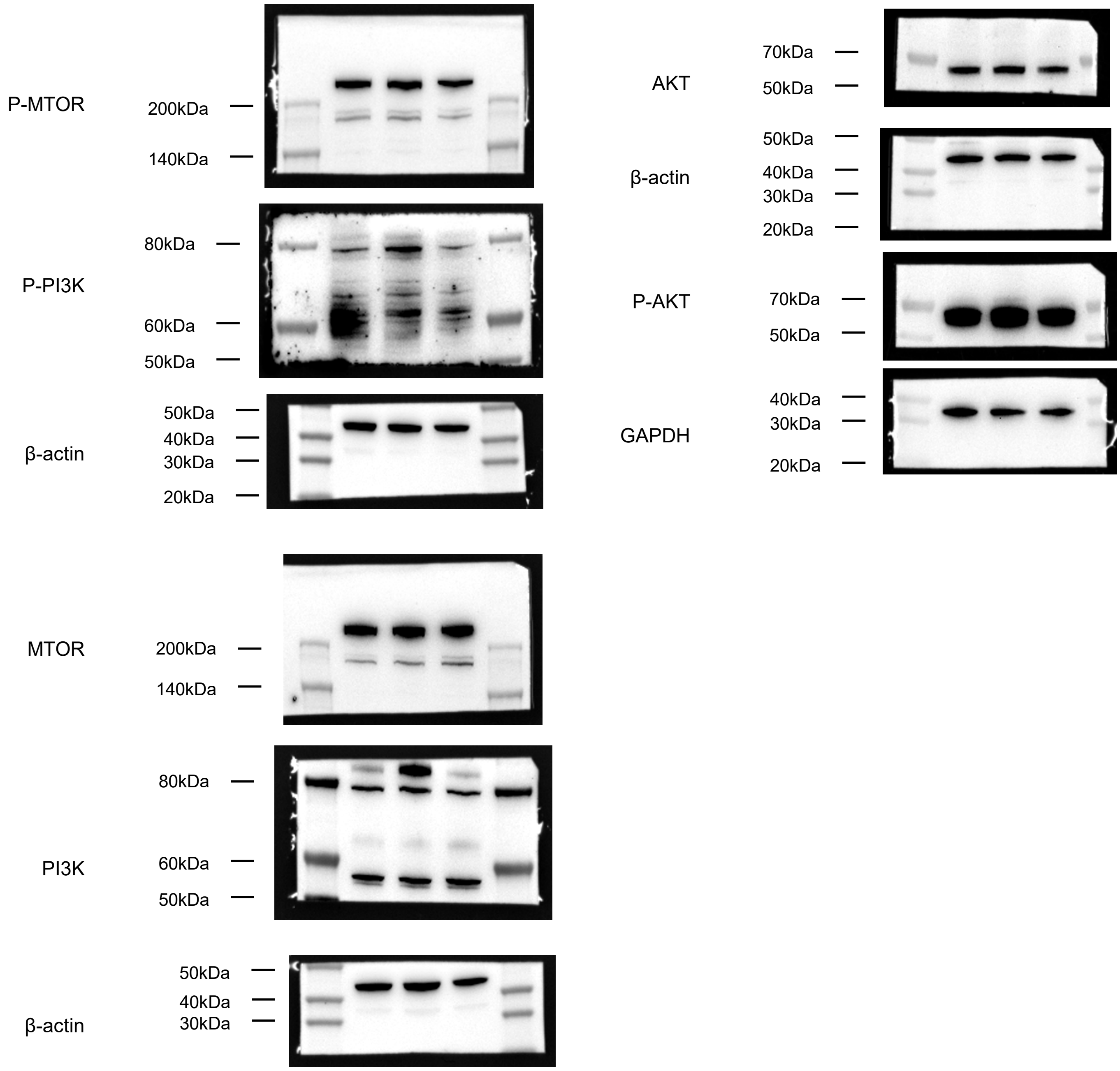

Fig. 6L Left

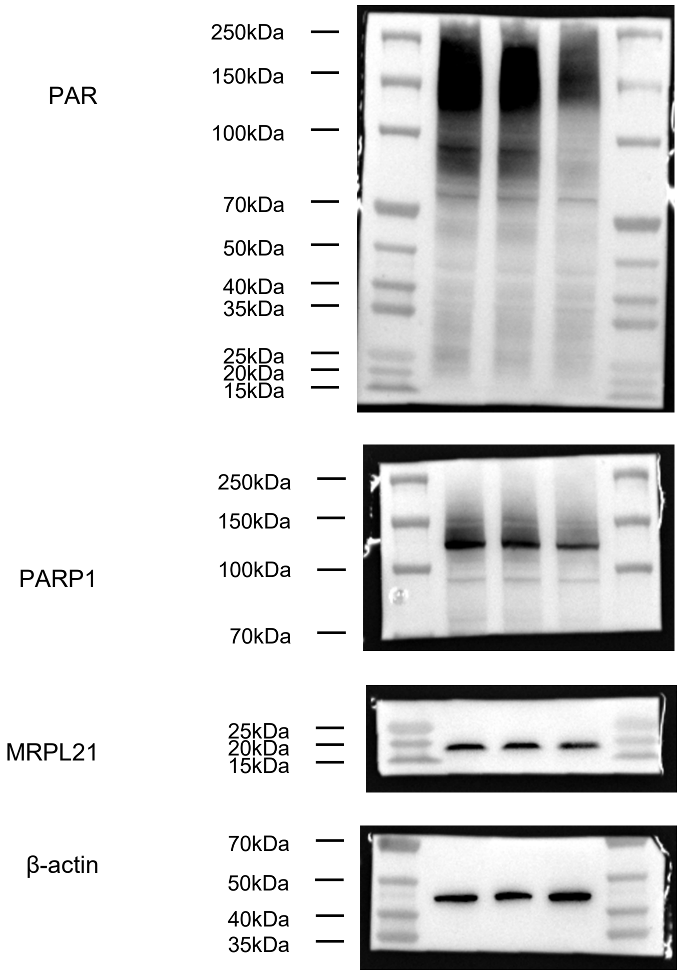

Fig. 6L Right

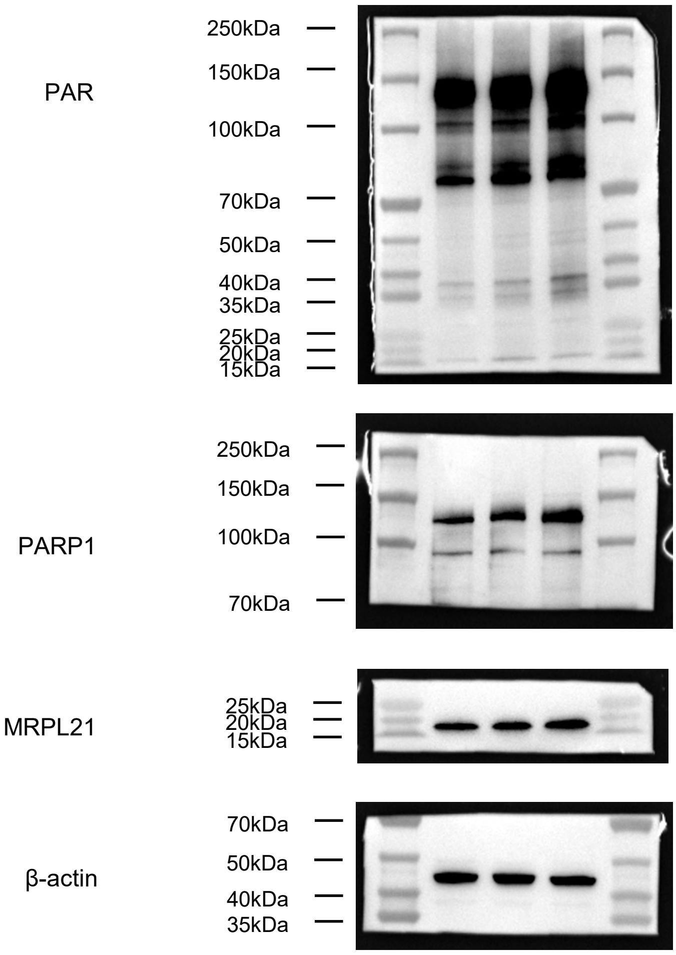

Fig. 6M

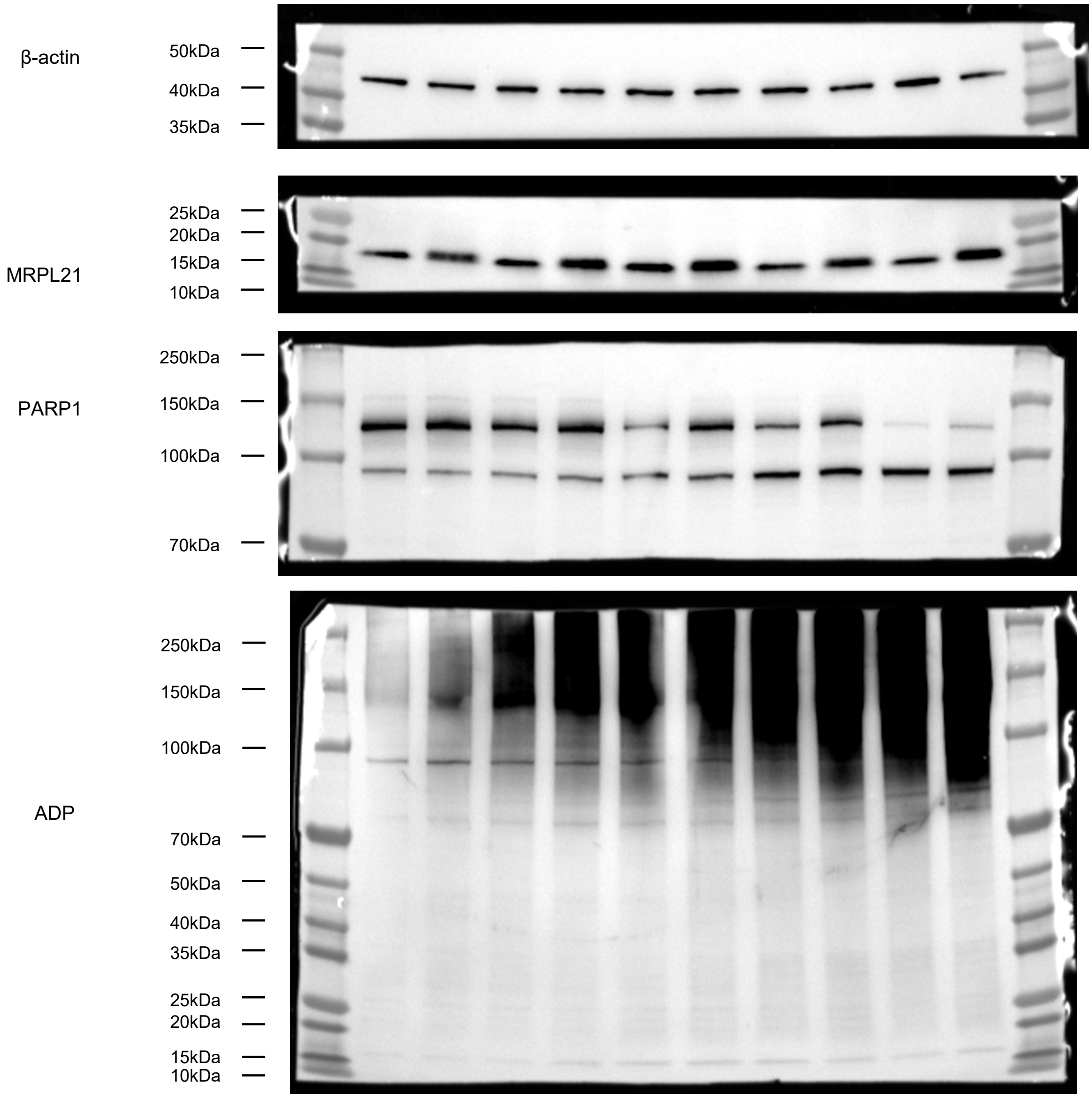

β-actin
50kDa
40kDa
35kDa
MRPL21
25kDa
20kDa
15kDa
10kDa
PARP1
250kDa
150kDa
100kDa
70kDa
ADP
250kDa
150kDa
100kDa
70kDa
50kDa
40kDa
35kDa
25kDa
20kDa
15kDa
10kDa

SFig. 2A

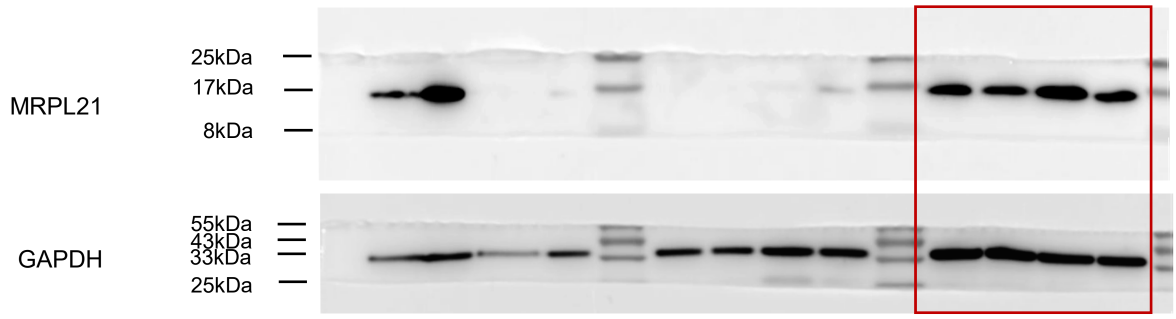

SFig. 3D

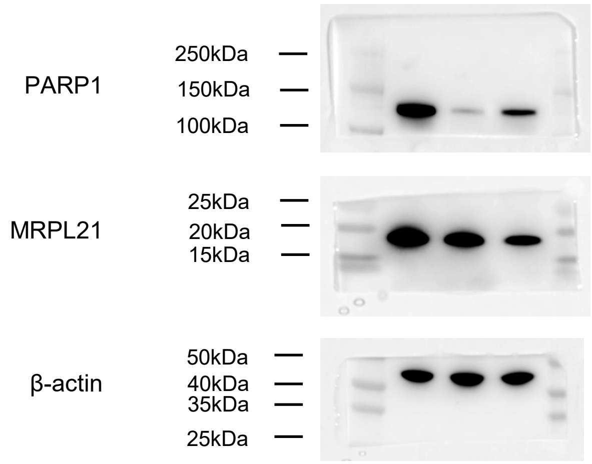

SFig. 5B

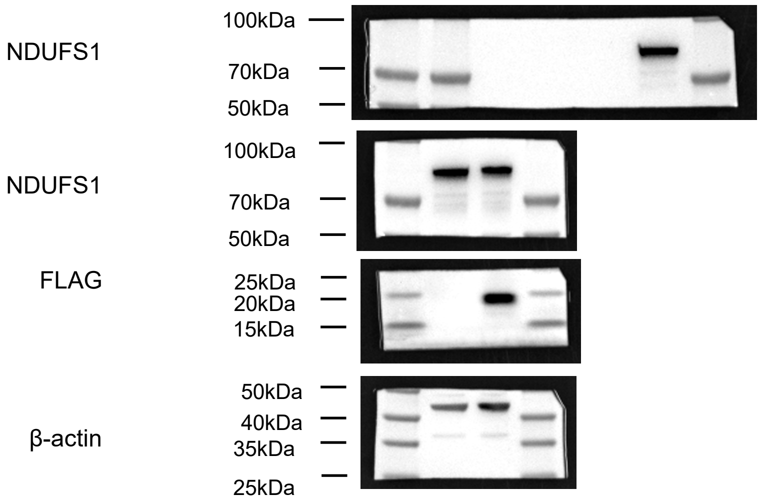

SFig. 5C

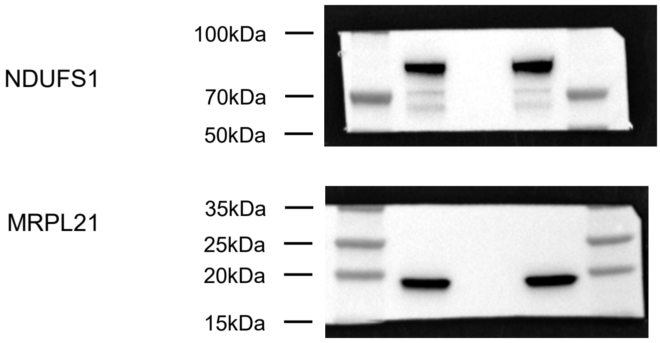

Supplement: Supplementary file 1 — Supplementary Material 1 [file 13046_2025_3482_MOESM1_ESM.zip › Western blot raw data.pdf]
