# Supplementary figures and images for: MRPL21-PARP1 axis promotes cisplatin resistance in head and neck squamous cell carcinoma by inhibiting autophagy through the PI3K/AKT/mTOR signaling pathway
Source: J Exp Clin Cancer Res. 2025 Jul 26;44:221. doi: 10.1186/s13046-025-03482-9 (PMC12297673; doi:10.1186/s13046-025-03482-9)

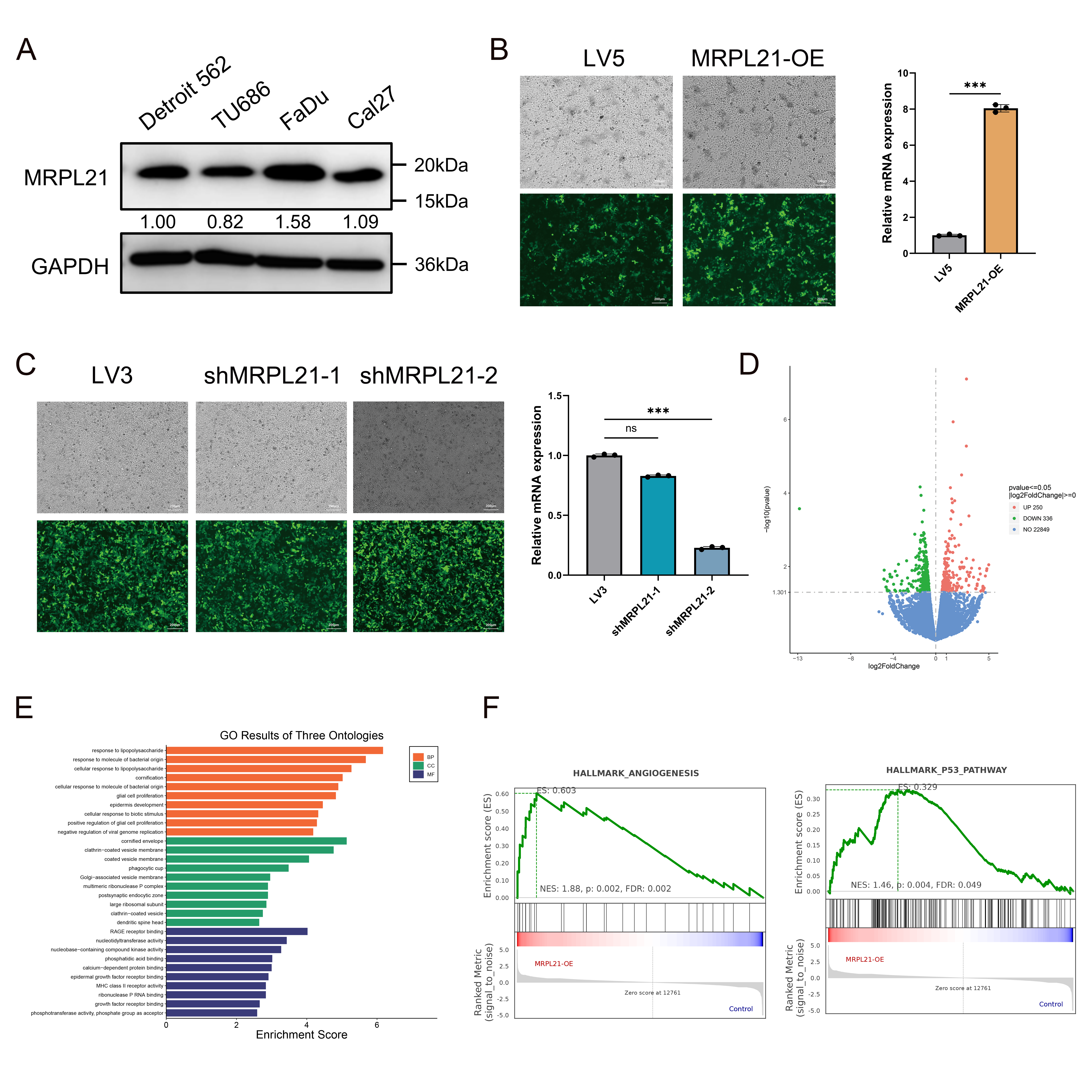

Supplement: Supplementary file 1 — Supplementary Material 1 [file 13046_2025_3482_MOESM1_ESM.zip › Supplementary figure 2.tif]
